# Circ0001470 Acts as a miR-140-3p Sponge to Facilitate the Progression of Embryonic Development through Regulating PTGFR Expression

**DOI:** 10.3390/cells11111746

**Published:** 2022-05-25

**Authors:** Long Zhang, Changfan Zhou, Xiaoyu Jiang, Shuntao Huang, Yiheng Li, Tao Su, Guowei Wang, You Zhou, Min Liu, Dequan Xu

**Affiliations:** 1 Colleges of Animal Science & Technology, Huazhong Agricultural University, Wuhan 430070, China; zlhzau.hzau.edu.cn@webmail.hzau.edu.cn (L.Z.); zhouchangfan@webmail.hzau.edu.cn (C.Z.); jxy.hzau.edu.cn@webmail.hzau.edu.cn (X.J.); shuntaohuang@webmail.hzau.edu.cn (S.H.); liyiheng@webmail.hzau.edu.cn (Y.L.); sutao@webmail.hzau.edu.cn (T.S.); wgw@webmail.hzau.edu.cn (G.W.); zhouyou_18@webmail.hzau.edu.cn (Y.Z.); liumin23@mail.hzau.edu.cn (M.L.); 2 Key Laboratory of Swine Genetics and Breeding of Ministry of Agriculture and Rural Affairs, Huazhong Agricultural University, Wuhan 430070, China; 3 Key Laboratory of Agricultural Animal Genetics, Breeding and Reproduction of Ministry of Education, Huazhong Agricultural University, Wuhan 430070, China; 4 College of Veterinary Medicine, Huazhong Agricultural University, Wuhan 430070, China

**Keywords:** circ0001470, miR-140-3p, endometrial epithelial cells, proliferation, embryonic development

## Abstract

Embryonic implantation and development are vital in early pregnancy and assisted reproduction. Circular RNAs (circRNAs) are involved in the two physiological processes and thus regulate animal reproduction. However, their specific regulatory functions and mechanisms remain unclear. Here, a novel circ0001470, originating from the porcine GRN gene, differentially expressed on day 18 versus day 32 of gestation in Meishan and Yorkshire pigs was screened. The circularization characteristic of circ0001470 was identified based on divergent primer amplification, Sanger sequencing, RNase digestion, and RNA nuclear-cytoplasmic fractionation. Functionally, circ0001470 can promote cell proliferation and cycle progression of endometrial epithelial cells (EECs) and also inhibit apoptosis of EECs using CCK-8 assays and flow cytometry analyses. Mechanistically, bioinformatics database prediction, luciferase screening, RNA immunoprecipitation (RIP), RNA-pull down, and FISH co-localization experiments revealed that the circ0001470 acted as a competing endogenous RNA (ceRNA) through sponging miR-140-3p to regulate downstream PTGFR expression. Moreover, in vivo assays revealed that mmu_circGRN promoted embryonic development by affecting the expression of PTGFR, which can activate the MAPK reproduction pathway and facilitate pregnancy maintenance. This study enriched our understanding of circRNAs in embryo implantation and development by deciding the fate of EECs.

## 1. Introduction

Sow litter size is an essential indicator reflecting the development of animal husbandry [1,2]. However, early embryo implantation and development are crucial factors limiting litter size [3,4]. Days 18 and 32 of pig pregnancy are the two peaks of embryonic loss [5,6]. Therefore, it is essential to explore the mechanism of endometrium changes and the genetic differences between different prolific breeds on days 18 and 32 of pig pregnancy. Notably, many studies have reported that the process of endometrium development is complex and involves numerous molecular mediators [7,8,9]. Although transcriptome profiling has identified several genes and non-coding RNAs that are aberrantly expressed during embryo implantation and development [10,11,12], little is known about the differences in circRNAs on the endometrium. 

CircRNAs, as a type of regulatory RNA, have attracted great research interest. CircRNAs are characterized by covalent closed-loop structures with neither 5′–3′ polarity nor a polyadenylated tail, which are abundant, conserved stable, and tissue or developmental-stage specific [13,14]. It has been described that circRNAs play essential roles in mammals’ reproduction. Such as the different temporal expression of circRNAs during pre-receptive and receptive embryonic implantation in dairy goats [15]. In human pre-implantation embryos, 10,032 circRNAs associated with embryonic development were identified by single-cell sequencing of PolyA-RNA [16]. The further study explored the expression pattern of circRNAs in mouse embryo implantation and development [17]. These findings are essential in future research into the potential roles of circRNAs in embryo implantation and development. 

Recently, ceRNA networks have been reported as an essential mechanism for explaining the post-transcriptional level of gene translation regulation [18]. Functioning as the ceRNA, circRNAs have been most widely reported in multiple physiological processes [19,20]. For example, circHIPK3 can directly bind to miR-124 and inhibit miR-124 expression levels and promote human cell proliferation [21]. CircTP63 acts as a ceRNA to regulate the expression of FOXM1 by sponging miR-873-3p, thereby affecting lung squamous cell carcinoma progression [22]. However, previous studies have not looked at the functions of ceRNA networks on EECs, and embryonic implantation and development remain largely unknown.

This study investigated the expression profiling of the circRNA-miRNA-mRNA in endometrium samples of Meishan and Yorkshire pigs on days 18 and 32 of pregnancy through whole transcriptome RNA-seq. Then, a significantly up-regulated circRNA in endometrial tissue of the two breeds at 32 days of pregnancy, designated as circ0001470, was initially identified. Originating from the exons 10 and 11 of the GRN gene, circ0001470 is stable and tissue-specific. By acting as a ceRNA to up-regulate PTGFR, circ0001470 promotes EECs proliferation, cell cycle progression, and induces apoptosis. Moreover, mmu_circGRN functions as a regulator during embryonic development in vivo. These findings help elucidate the molecular regulation in EECs and may provide essential information to support further research on the development of the endometrium.

## 2. Materials and Methods

### 2.1. Animal Sources and RNA Sequencing

The experimental population included four Yorkshire and four Meishan sows. All pigs were raised under the same conditions, with a standardized feeding regimen and free access to water. On days 18 and 32, two Yorkshire sows and two Meishan sows were slaughtered at each time-point, and the endometria were taken [23]. In our research, all animal experiments were conducted according to the Care and Use of Laboratory Animals Guidelines in China. All animal procedures were approved by the Scientific Ethics Committee of Huazhong Agricultural University (ID number: HZAUSW2015-017, permitted in February 2015). 

RNA-seq analysis was performed on the endometrial tissues. Total RNA was extracted from the endometrial samples using TRIzol reagent (Invitrogen, Life Technologies, Pleasanton, CA, USA) according to the manufacturer’s instructions. Ribo-Zero Magnetic Kit (Epicenter, Chicago, IL, USA) was used to deplete ribosomal RNA. The HISAT2 package mapped the raw reads to the Sus scrofa reference genome (version 10.2). The expression level of mRNAs was quantified as Transcripts Per Kilobase Million (TPM) using the StringTie package. The number of gene reads was collected using HTSeq software (0.6.1) according to the Sus scrofa GTF file (version 10.2). Subsequently, DEGs were identified using the R packages DEGseq (1.18.0). In addition, cluster analysis was applied to differentially express circRNAs among the four groups (YK18, YK32, MS18, MS32) as shown in Supplementary Figure S1. The RNA-seq data were stored in the Gene Expression Comprehensive Repository (GSE141564).

### 2.2. Identification and Verification of circRNAs

CIRCexplorer software was used to identify circRNAs, with at least two raw reads across its junction region. Differentially expressed circRNAs were identified by the edgeR software package, with cutoffs of |log10(FC)| ≥ 1, *p* ≤ 0.05, and FDR ≤ 0.5, where “FC” indicated fold-change in expression for the gene. The differentially expressed circRNAs are shown in Supplementary Text S1.

### 2.3. Real-Time Quantitative PCR (qRT-PCR) for Gene Expression, RNase R Treatment, and Actinomycin D Assay

Total RNA was extracted with TRIzol reagent (Invitrogen, Life Technologies, Pleasanton, CA, USA). Reverse transcription of miRNAs and mRNAs was conducted using RevertAid first-strand cDNA (TaKaRa, Dalian, China) according to the manufacturer’s instructions. qRT-PCR was performed using SYBR Green PCR Master Mix (Vazyme, Nanjing, China) in a 10 μL reaction and the Roche LightCyler 480 system (Roche, Germany), with porcine β-actin gene as the reference for circRNA, mRNA, and U6 for miRNA. All primers for qRT-PCR are shown in Supplementary Table S1, and the relative expression levels were calculated using the equation N = 2^−ΔΔCt^. The relative fold changes in mRNA expression levels were quantified by normalizing the experimental gene’s cycle threshold (CT) value to the mean CT value of the control β-actin gene. 

Another total RNA was incubated for 15 min at 37 °C with and without (mock) 3 U/μg of RNase R (Epicenter Biotechnologies, Chicago, IL, USA), respectively. To quantify the number of circRNAs, we synthesized cDNA from random hexamers using the Eraser gDNA Prime Script RT kit (TaKaRa, Dalian, China). qRT-PCR was used to determine the abundance of circRNA and the divergent primers sets are listed in Supplementary Table S1. The PCR products from the individual circRNAs were semi-quantitatively analyzed in 3% agarose gel electrophoresis and further identified by Sanger sequencing.

Total RNA from EECs was treated with 100 ng/mL actinomycin D (Cell Signaling Technology, Beverly, MA, USA) for 4 h, 8 h, 12 h, and 24 h, respectively. Then, RNA was reversed and transcribed into cDNAs with the PrimeScript RT Reagent Kit (Takara, Dalian, China) according to the manufacturer’s instructions. qRT-PCR was performed using TB Green Premix Ex Taq (Takara, Dalian, China). Relative levels of gene expression were determined with the 2^−ΔΔCt^ method.

### 2.4. Vector Construction and Small Fragment Synthesis

MiR-140-3p binding sequences in circ0001470 and PTGFR were identified using RNAhybrid software. To construct reporters for luciferase assays, the wild psiCHECK2-circ0001470-WT (WT-circ0001470) was generated by inserting the circ0001470 fragments full length into the psiCHECK-2 vector (Promega, Madison, WI, USA) at the 3′end of Renilla gene. The mutant psiCHECK2-circ0001470-MUT (MUT-circ0001470) was generated by mutating the complementary to the seed region of the miR-140-3p using overlapping extension PCR. To construct reporters, 3′-UTR of PTGFR containing the miR-140-3p target site was cloned and inserted downstream of the Renilla luciferase gene in the psiCHECK-2 vector, and the mutated plasmids were constructed. All constructs were verified by sequencing. The primers are shown in Supplementary Table S2.

To construct vectors for overexpression assays, the full length of circ0001470 was cloned into the pCD2.1-ciR vector (Geneseed Biotech, Guangzhou, China). The primers are shown in Table S2, and the sequences are shown in Supplementary Table S3. The coding domain sequence (CDS) of PTGFR was cloned and inserted in the pcDNA3.1(+) vector (Promega, Madison, WI, USA). All constructs were verified by sequencing. 

Two pairs of specific interference fragments of circ0001470 (si-circ0001470-1, si-circ0001470-2) were designed based on the back-splice junction of circ0001470 (Geneseed, Guangzhou, China). The miR-140-3p mimic and miR-140-3p inhibitor fragments were synthesized according to the porcine reference sequence (GenePharma, Shanghai, China). Two pairs of specific interference fragments of PTGFR (si-PTGFR-1, si-PTGFR-2) were designed using the siRNA Design Software according to the protein-coding sequence of PTGFR of the porcine reference genome (GenePharma, Shanghai, China). The interference fragments are shown in Supplementary Table S4.

### 2.5. EECs Isolation, Culture, and Transfection

The intermediate parts of the uterine horns harvested from the sow were opened longitudinal under aseptic conditions. The endometrium tissues were collected and flushed with PBS (pH = 7.4) (Hyclone, Logan, UT, USA) and placed in a culture medium, with 0.1% collagenase I mixed with Dulbecco’s modified eagle medium/Nutrient mixture F-12 (DMEM/F-12) (Hyclone, Logan, UT, USA). The mixture was placed in a 5% CO_2_ incubator at 37 °C for 2.5 h and shaken every half hour. The DMEM/F-12 culture medium containing 10% fetal bovine serum (FBS) (FBS, Gibco, Brooklyn, NY, USA) supplemented with 1% penicillin-streptomycin was used to terminate the digestion. Afterward, the mixture was filtered using the sterilized cell screen, and the solution was transferred to the tube. The sediments were collected to release the endometrial cells after centrifugation twice. The differential adhesion method was used to isolate EECs and endometrial stromal cells (ESCs). The isolated EECs were cultured in Dulbecco’s modified Eagle’s medium/F-12 (DMEM/F12; 1:1) medium containing 10% fetal bovine serum and 1% penicillin-streptomycin at 37 °C under 5% CO_2_ in humidified air. 

The purified EECs were plated and grown in 6-well plates. Then transfected at 75% confluency with overexpression treatment (circ0001470 + pCD2.1-ciR or miR-140-3p mimic or PTGFR + pcDNA3.1+) and small interfering RNAs (si-circ0001470 or miR-140-3p inhibitor or si-PTGFR) using lipofectamine 2000 (Invitrogen, Carlsbad, CA, USA) according to the manufacturer’s protocols, respectively. 

### 2.6. Luciferase Assay

The wild-type (WT-circ0001470/WT-PTGFR) or mutated (MUT-circ0001470/MUT-PTGFR) plasmids were co-transfected with the miR-140-3p mimic or inhibitor into PK15 cells, respectively. After 24 h post-transfection, firefly and Renilla luciferase activities were measured with the Dual-Glo luciferase assay system (Promega, Madison, WI, USA). Each test was performed three times.

### 2.7. Protein Extraction and Western Blot Analysis

After being transfected with plasmids and siRNAs for 48 h, the cells were split by radioimmunoprecipitation assay (RIPA) buffer (Servicebio, Wuhan, China) and supplemented with 0.01% of phenylmethanesulfonyl fluoride (PMSF) (Servicebio, Wuhan, China), then harvested and lysed in RIPA buffer at 4 °C for 30 min and centrifuged (12,000 *g*) for 10 min. Soluble proteins in the supernatants were collected. Total protein concentrations were determined using the Bio-Rad DC Protein Assay (Hercules, CA, USA) after diluting with 80 μL 5× protein loading buffer (Servicebio, Wuhan, China) and boiling for 10 min. The proteins were separated by polyacrylamide gel electrophoresis on sodium dodecyl sulfate (SDS) and then applied to polyvinylidene fluoride (PVDF) membranes (Millipore, Boston, MA, USA). Next, the proteins were incubated with the corresponding primary and secondary antibodies. The antibodies used are listed in Supplementary Table S5. Western blot analysis was performed using Quantity One software (Bio-Rad, Hercules, CA, USA). 

### 2.8. RNA FISH and RNA Nuclear-Cytoplasmic Fractionation

Cy3-labeled circ0001470 and FITC-labeled miR-140-3p probes (Geneseed, Guangzhou, China) were applied to observe the location of circ0001470 and miR-140-3p in EECs. After pre-hybridization at 55 °C for 2 h, cell climbing slides were hybridized with a specific Cy3-labeled circ0001470 probe and FITC-labeled miR-140-3p probe at 37 °C overnight, and cell nuclei were stained with 4,6-diamidino-2-phenylindole (DAPI, Beyotime, Shanghai, China). The slides were photographed under a fluorescence microscope (Leica, Wetzlar, Germany). Cy3-labeled circ0001470 and FITC-labeled miR-140-3p probes sequences are shown in Supplementary Table S4.

RNAs from the nucleus and cytoplasm of EECs were separated by the PARIS™ Kit (Life Technologies, Austin, TX, USA) following the manufacturer’s instructions.

### 2.9. Immunohistochemistry (IHC)

The tissue slides were dissociated in xylene and dehydrated in diminishing ethanol concentrations, then subjected to antigen retrieval in sodium citrate buffer for 10 min in a 100 °C microwave oven. The endogenous peroxidase was inactivated by incubation with 3% hydrogen peroxide at room temperature for 10 min. Tissue slides were blocked for 30 min and incubated overnight at 4 °C with primary antibody of PTGFR (1:200; ab188935). The tissue slides were then incubated with biotinylated secondary antibodies for 60 min at 37 °C. After washing for 10 min in TBS, Diaminobenzidine (DAB; Zytomed Systems, Berlin, Germany) according to the manufacturer’s instructions was used to dye tissue slides. Finally, the slides were washed under running tap water for 5 min and counterstained with hematoxylin (Servicebio, Wuhan, China). Images were taken by an Olympus multifunction microscope (Olympus BX51, Tokyo, Japan).

### 2.10. RNA Immunoprecipitation (RIP)

RIP experiments were carried out using the Magna RIP RNA-Binding Protein Immunoprecipitation Kit (Millipore, Billerica, MA, USA) according to the manufacturer’s instructions. Briefly, The Ago2-RIP assay was conducted in approximately 1 × 10^7^ EECs. The cells were pelleted and re-suspended with an equal pellet volume of 100 mL RIP Lysis Buffer plus protease and RNase inhibitors. Then the cell lysates were incubated with an anti-AGO2 antibody or IgG negative control antibody at 4 °C overnight. Subsequently, cells were lysed and incubated with appropriate antibody-coated beads. The immunoprecipitated RNAs were extracted by RNeasy MinElute Cleanup Kit (Qiagen, Hilden, Germany) and reversely transcribed using PrimeScript RT Master Mix (TaKaRa). The abundance of circ0001470 was detected by qRT–PCR assay.

### 2.11. Biotin-Coupled Probe RNA Pull-Down Assay

The pull-down assay with biotinylated miRNA was performed as previously described [24]. Briefly, the 3′-end biotinylated miR-140-3p mimic or control RNA (50 nM, Ribobio, Guangzhou, China) was transfected into approximately 1 × 10^7^ EECs, before transfection of the WT-circ0001470 or the MUT-circ0001470. After that, the biotin-coupled RNA complex was incubated with the biotin-labeled miR-140-3p probe and then lysed. The cell lysate was incubated overnight at 4 °C with the probe-coated bead mix. After washing with wash buffer, the precipitated RNA complexes were eluted and purified with TRIzol reagent (Takara, Kyoto, Japan). The abundance of circ0001470 was detected by qRT-PCR.

### 2.12. CCK-8 Assay 

For the EECs proliferation assay, 3 × 10^3^ cells were incubated in 100 μL of complete culture media in 96-well plates for different durations (12 h, 24 h, 48 h, 72 h). Cell Counting Kit-8 reagent (Dojindo Laboratories, Kumamoto, Japan) was added to each well and incubated at 37 °C for 2 h. An automatic microplate reader measured the optical density at 450 nm (Bio-Rad, CA, USA).

### 2.13. 5-Ethynyl-20-Deoxyuridine (EdU) Incorporation Assay

The proliferation activity of EECs was tested by EdU kit (BeyoClick™ EdU Cell Proliferation Kit with Alexa Fluor 488, Beyotime, Shanghai, China). Briefly, EECs (2 × 10^4^ cells/well) were seeded in 24-well plates and cultured in support or interference clips for 72 h. The cells were then incubated with EdU for 3 h, fixed with 4% paraformaldehyde for 15 min, and permeabilized with 0.3% Triton X-100 for 15 min. Next, the cells were incubated with Click Reaction Mix for 30 min at room temperature in the dark, followed by Hoechst 33342 for 10 min. Finally, the dyed EECs were photographed and counted under an Olympus FV1000 microscope (Olympus, Tokyo, Japan). Each experiment was repeated three times in triplicate.

### 2.14. Cell Cycle Assay

EECs (2 × 10^6^) were harvested after transfection for 48 h. After washing twice with cold PBS, the cells were fixed with 90% ethanol at 4 °C overnight and re-washed twice to remove the ethanol. Then, the EECs were mixed with100 μL RNase A (Servicebio, Wuhan, China) at 37 °C for 40 min. The cells were then stained with 400 μL propidium iodide (Servicebio, Wuhan, China) for 20 min. FACS was applied to evaluate the ratio of cells in different phases at 488 nm, and ModFit LT software(Cell Quest) was used for analysis. Each experiment was performed three times.

### 2.15. Cell Apoptosis Assay

EECs apoptosis was measured by the flow cytometry method (FCM) and the Annexin V-FITC PI staining apoptosis assay kit (Servicebio, Wuhan, China) according to the manufacturer’s protocol. Briefly, Annexin V-positive and PI-negative cells were prepared in 20 µL binding buffer, followed by the treatment in 10 µL Annexin V-FITC and 5 µL PI. Each experiment was performed three times. The percentage of apoptotic cells was detected by flow cytometry (Accuri C6II, BD, New Yock, NJ, USA). The data were analyzed using FlowJo v7.6 software.

### 2.16. Lentiviral Vector Construction

To generate mmu-circGRN overexpression lentiviral plasmid, circGRN was constructed into a pHBLV plasmid. pHBLV-circGRN, PsPAX2, and pMD2G plasmids were co-transfected into HEK293T cells for virus packaging (LV-mmu_circGRN). Then pHBLV, PsPAX2, and pMD2G plasmids were co-transfected into HEK293T cells for virus packaging (LV-mmu_NC) The mouse circGRN sequences are shown in Supplementary Table S6.

### 2.17. Uterine Horn Injection of Mice

It is challenging to conduct in vivo tests in pigs, so we used six mice as the mouse implantation model after referring to other studies which focused on pig implantation [25]. The day of discovery of the vaginal suppository after intercourse is specified as the first day of pregnancy. The uterine horn injection was conducted on ten mice under general anesthesia on the evening of the third day of pregnancy. Ten microliters of 10^8^ TU/mL LV-mmu_NC and LV-mmu_circGRN were injected into the left and right uterine horns, respectively. The wound was then sutured, and the mice were placed in a warm environment at 37 °C until they awoke from anesthesia. Analgesics were delivered to mice at 12, 24, and 48 h after surgery to relieve pain. Three mice were killed on day five, and the others were killed on day 10. Endometrial tissues were collected, embryos were detached, and the numbers of implanted embryos were counted by visual observation. Endometrial tissue was ground to extract RNA to detect lentiviral infection efficiency and downstream target gene expression levels.

### 2.18. Statistical Analysis

The results are represented as mean ± standard error of the mean (SEM), and analyses were performed using SPSS 17.0 (SPSS Inc., Chicago, IL, USA) and Graphpad 7.0 software. Differences were compared using one-way ANOVA, and the least significant difference (LSD) method was used for further analysis. A two-tailed *p* < 0.05 is considered a significant difference and highly effective at *p* < 0.01 or *p* < 0.001. All data are from at least three independent experiments.

## 3. Results

### 3.1. Circ0001470 Is Upregulated in Endometrial Tissue during Embryonic Development

**Figure 1:**
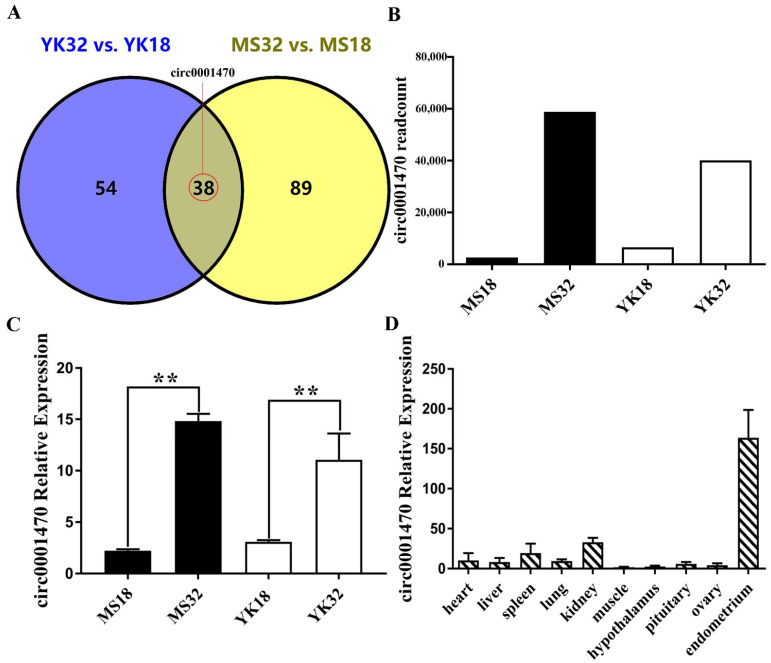
Circ0001470 expression profiling was screened and identified in endometrial tissue. (**A**) Venn diagram analysis of the number of differentially expressed circRNAs during the different periods of the Meishan and Yorkshire pigs. (**B**) RNA-seq sequencing data revealed a readcount of circ0001470 in the endometrium of the two breeds at 18 and 32 days of gestation. (**C**) Circ0001470 expression patterns in the endometrium of Meishan and Yorkshire pigs at 18 and 32 days of gestation. (**D**) Circ0001470 expression levels in different tissues of Yorkshire pigs at 32 days of gestation were detected by qRT-PCR. The data are represented as mean ± standard deviation. All data are representatives of three independent experiments. ** represents *p* < 0.01.

To identify circRNAs differentially expressed during the critical period of embryo loss in the two breeds, 38 differentially expressed circRNAs were found in endometrial tissues from the two breeds at two gestations (Figure 1A). We focused on a novel circRNA, which we named circ0001470, that is differentially expressed during different pregnancy days of the two breeds according to RNA-seq data (Figure 1B). Then, we validated the expression level of circ0001470 using qRT-PCR. The expression levels of circ0001470 were consistent with the RNA-seq data and upregulated in endometrial tissue at 32 days of pregnancy (Figure 1C). Moreover, circ0001470 is expressed highly in endometrial tissue (Figure 1D). Therefore, we speculate that circ0001470 may affect embryonic development.

### 3.2. The Characteristics of circ0001470 in EECs

**Figure 2:**
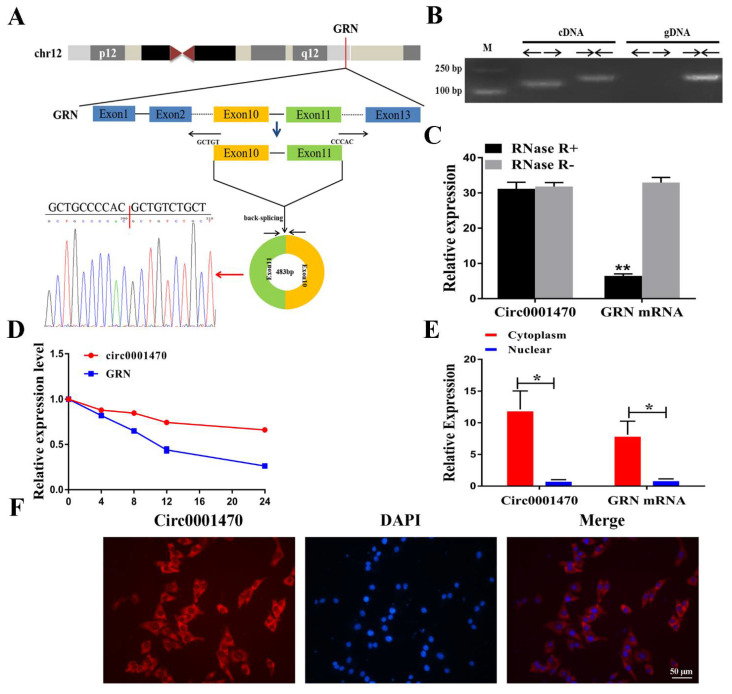
Characterization of circ0001470 RNA in EECs. (**A**) A sketch of the structure of circ0001470, which was generated from an exon10 and exon11 region of the GRN gene via back-splicing. (**B**) PCR analysis for circ0001470 and linear isoform GRN in cDNA and genomic DNA (gDNA). (**C**) Resistance of circ0001470 and linear transcript GRN by qRT-PCR after RNase enzyme treatment of EECs. (**D**) qRT-PCR for the abundance of circ0001470 and GRN in EECs treated with Actinomycin D at the indicated time points. (**E**) qRT–PCR data indicated the abundance of circ0001470 and GRN mRNA in the cytoplasm and nucleus of EECs. The circ0001470 and GRN mRNA levels were normalized to the value measured in the cytoplasm. (**F**) The localization of circ0001470 was detected by FISH. Circ0001470 was labeled by red fluorescence. Nuclei were stained with 4,6-diamidino-2-phenylindole (DAPI). Scale bar, 50 μm. Data are presented as mean ± standard deviation. All data are representatives of three independent experiments. * *p* < 0.05, ** *p* < 0.01.

Originating from the GRN gene, circ0001470 is located at chromosome 12 and contains head-to-tail splicing of exons 10 and 11. The circular structure of circ0001470 was confirmed by amplification with divergent primers followed by Sanger sequencing (Figure 2A). However, head-to-tail splicing may not only arise from trans-splicing but also from genome rearrangement, and PCR analysis was used to rule out the latter. Special divergent primers were designed to amplify circ0001470. cDNA and gDNA were extracted separately from EECs and were subjected to PCR and agarose gel electrophoresis assays (Figure 2B). The results showed that circ0001470 was detected only in cDNA, with no products in the gDNA. Furthermore, the expression levels of post-spliced or canonical forms of GRN with or without RNase R in EECs cDNA and gDNA were examined by qRT-PCR (Figure 2C). Circ0001470 was detected only in cDNA after divergent primers amplification and even when treated with RNase R. Additionally, the primers failed to amplify the linear form of GRN, suggesting that circGRN is not caused by genomic rearrangements or PCR products. We then examined the stability and localization of circ0001470 in EECs. After the transcription inhibitor actinomycin D treatment, total RNA was collected at indicated time points. An analysis of circ0001470 and GRN mRNA revealed that circ0001470 was highly stable, with a half-life exceeding 24 h. In contrast, the associated linear transcript exhibited a half-life of less than 4 h (Figure 2D). qRT–PCR analysis of nuclear and cytoplasmic circ0001470 RNA and FISH assay demonstrated that circ0001470 preferentially localized in the cytoplasm (Figure 2E,F). Taken together, our results indicate that circ0001470 is an abundant and stable circRNA expressed in EECs.

### 3.3. Circ0001470 Promotes EECs Proliferation, Cycle Transition, and Suppresses Apoptosis

**Figure 3:**
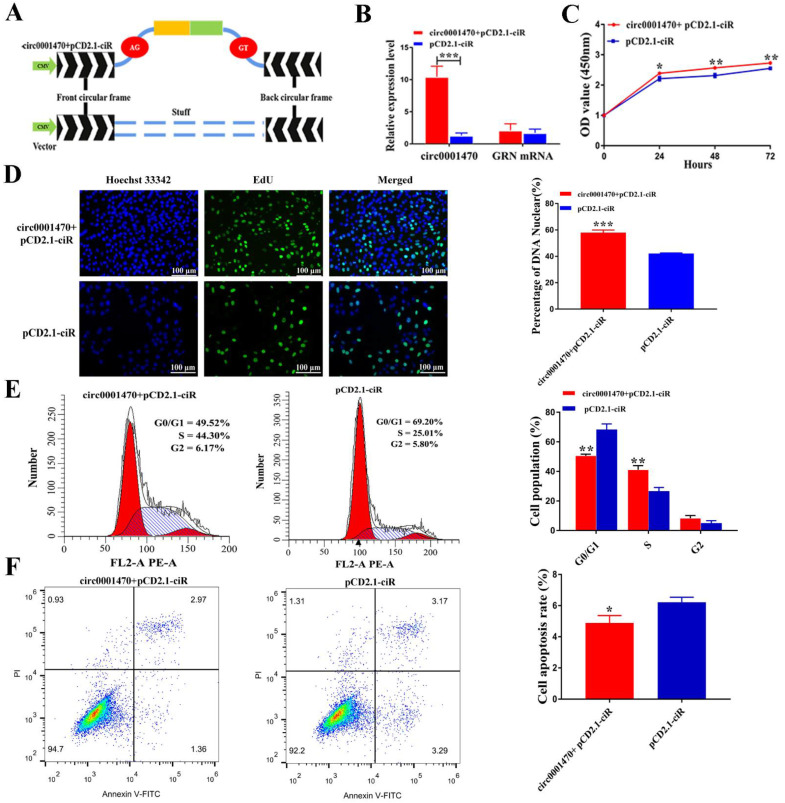
Circ0001470 promotes EECs proliferation, and cycle transition and suppresses apoptosis. (**A**) Schematic illustration of circ0001470 expression vector. (**B**) qRT-PCR analysis of circ0001470 and GRN mRNA in EECs after overexpression circ0001470. (**C**) CCK-8 demonstrated the proliferation ability of EECs transfected with circ0001470 or the vector. (**D**) The proliferation of EECs transfected with circ00001470 overexpression plasmids or vector treatment was detected by EdU assays. Scale bars, 100 μm. (**E**) Cell cycle distribution was measured in EECs transfected with circ0001470 or vector. (**F**) FCM analysis shows the ratios of apoptotic EECs with the treatment of circ0001470 or vector. Data are shown as mean ± SD; All data are representatives of three independent experiments. * *p* < 0.05, ** *p* < 0.01, *** *p* < 0.001.

To explore the biological function of circ0001470 in EECs, EECs were purified by collagenase I digestion of endometrial tissue, followed by differential centrifugation (Supplementary Figure S2A). The overexpression vector of circ0001470 was successfully constructed (Figure 3A), as it increased circ0001470 expression level without influencing GRN mRNA level (Figure 3B). Two siRNAs were designed to silence circ0001470 without affecting the GRN mRNA level in EECs (Supplementary Figure S2B,C). Finally, si-circ0001470-1 was chosen for the following experiment due to its high inhibitory efficiency.

CCK-8 assays demonstrated that the upregulation of circ0001470 significantly enhanced the cell proliferation viability. In comparison, the downregulation of circ0001470 had the opposite effect (Figure 3C; Supplementary Figure S2D). Additionally, EdU assays revealed that overexpression of circ0001470 significantly increased the percentages of EdU-positive cells, which considerably decreased when knocked down circ0001470 (Figure 3D; Supplementary Figure S2E). Changes in proliferation might be caused by an aberrant cell cycle. Therefore, the effect of circ0001470 on cell cycle progression was measured by FACS analysis, and the results showed that circ0001470 overexpression led to fewer cells arrested in the G0/G1 phase and more cells in the S and G2/M phase. In addition, the knockdown of circ0001470 showed the opposite results (Figure 3E; Supplementary Figure S2F), suggestingcirc0001470 silencing suppressed the proliferation and cell cycle of EECs. Besides, circ0001470 was verified to regulate apoptosis of EECs, and the upregulation of circ0001470 was significantly attenuated in apoptotic cells. In contrast, EECs transfected with circ0001470 siRNA showed a significant increase in the ratio of apoptotic cells compared to the si-NC group (Figure 3F; Supplementary Figure S2G). These experiments suggest that circ0001470 promoted proliferation, cycle, and induced apoptosis of EECs.

### 3.4. Circ0001470 Directly Targeted miR-140-3p

**Figure 4:**
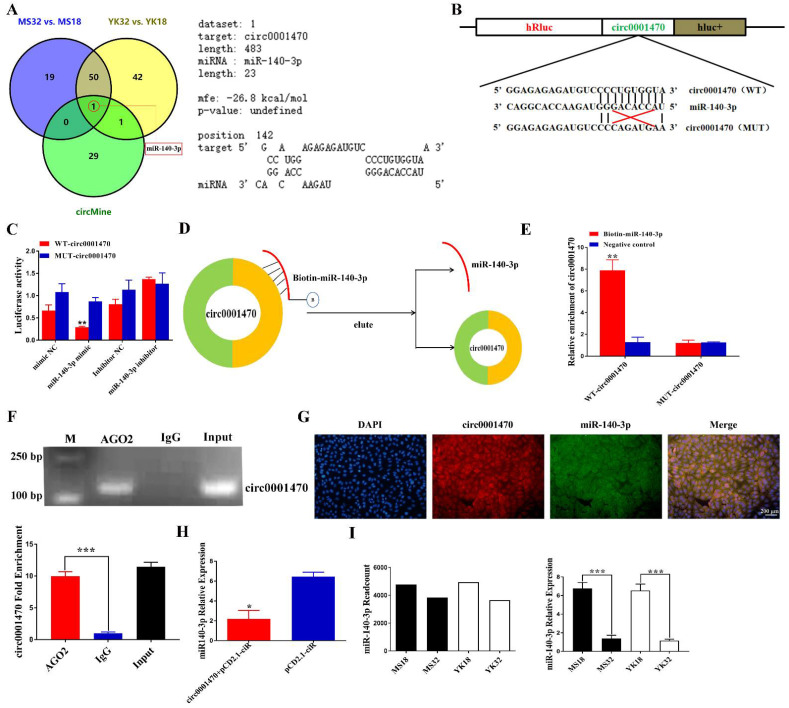
Circ0001470 decreased miR-140-3p levels, serving as a microRNA sponge in EECs. (**A**) Venn diagram demonstrating the possible binding of circ0001470 to differentially expressed miRNAs in endometrial tissues of pigs at different gestation days. An analysis of the binding free energy of circ0001470 to miR-140-3p by RNAhybrid database. (**B**) Schematic diagram showing the design of luciferase reporters with the WT-circ0001470 and the site-directed MUT-circ0001470, respectively. ‘TGTGGT’ is the ‘seed sequence’ of miR-140-3p; mutant nucleotide is ‘CAGATG’. (**C**) Luciferase assay was conducted after transfection, WT-circ0001470 or its mutant luciferase reporter vectors were co-transfected with miR-140-3p mimic or mimic NC (miR-140-3p inhibitor or inhibitor NC) into PK15 cells. (**D**) Schematic diagram displaying the pull-down of circ0001470 by a biotin-labeled miR-140-3p probe. (**E**) RNA pulldown assay was performed with biotin-miR-140-3p, control or WT-circ0001470 or MUT-circ0001470 by using EECs extracts. RNA levels of miR-140-3p in immunoprecipitates were detected by qRT-PCR. (**F**) AGO2 RNA immunoprecipitation assay of circ0001470 in EECs. Semi-quantitative PCR and qRT- PCR results of AGO2 pull-down circ0001470. (**G**) The cellular position of circ0001470 (red) and miR-140-3p (green) in cells was observed by FISH (scale bars, 200 μm). (**H**) qRT-PCR analysis of miR-140-3p expression in EECs treated with overexpression circ0001470. (**I**) The miR-140-3p levels in Yorkshire and Meishan endometrium at 18 and 32 days of pregnancy. Data are presented as the mean (n = 3) standard deviation. Student’s *t*-test; * *p* < 0.05, ** *p* < 0.01, *** *p* < 0.001.

Circ0001470 is mainly localized in the cytoplasm, so we speculate that circ0001470 functions by sponging miRNAs and regulating miRNA expression. circMine database was used to screen candidate miRNAs targeted by circ0001470 as shown in Supplementary Text S2. Afterward, miR-140-3p was screened by joint analysis on differentially expressed miRNAs during different pregnancy periods of the two breeds (Figure 4A; left panel). Furthermore, an analysis of the RNAhybrid database revealed that circ0001470 has a binding relationship with the seed sequence of miR-140-3p, with the free energy of the binding site as high as -26.8 kcal/mol (Figure 4A; right panel). 

To determine whether circ0001470 directly targets miR-140-3p through the predicted binding sites, psiCHECK-2 reporter and mutant plasmids were constructed (Figure 4B). The results indicated the luciferase activity of the wild-type circ0001470 (WT-circ0001470) group significantly decreased in the miR-140-3p mimic group and increased in the miR-140-3p inhibitor group compared with the inhibitor negative control group. In addition, this reduction was not observed in the mutant plasmids (MUT-circ0001470) (Figure 4C). To further confirm the direct interaction between circ0001470 and miR-140-3p, RNA pull-down assay with a biotin-labeled miR-140-3p probe was applied (Figure 4D). The results of qRT-PCR showed that the biotin-labeled miR-140-3p probe captured more WT-circ0001470 than the negative control probe did. In addition, the biotin-labeled miR-140-3p probe cannot be enriched by MUT-circ0001470 (Figure 4E). 

It is widely believed that miRNAs regulate the expression of target genes by binding to Argonaute 2 (AGO2), an essential component of the RNA-induced attenuation complex (RISC). Therefore, an RNA immunoprecipitation (RIP) experiment was conducted by using anti-AGO. The agarose gel electrophoresis and qRT-PCR detection showed that both anti-AGO2 and circ0001470 could be effectively pulled down compared to the control, and circ0001470 has been found to preferentially accumulate in AGO2 containing miRNPs (Figure 4F). Moreover, confocal data indicated that circ0001470 and miR-140-3p were co-localized in the cytoplasm (Figure 4G). In addition, the overexpression of circ0001470 in EECs significantly reduced the expression level of dissociative miR-140-3p (Figure 4H). Furthermore, miRNA-seq and qRT-PCR present evidence that the expression level of miR-140-3p is higher at 18 days of pregnancy than at 32 days of pregnancy in both breeds (Figure 4I), whereas circ0001470 has an opposite expression pattern in the endometrial tissue. Collectively, these data demonstrated that circ0001470 acted as a sponge for miR-140-3p.

### 3.5. miR-140-3p Directly Targets PTGFR in EECs

**Figure 5:**
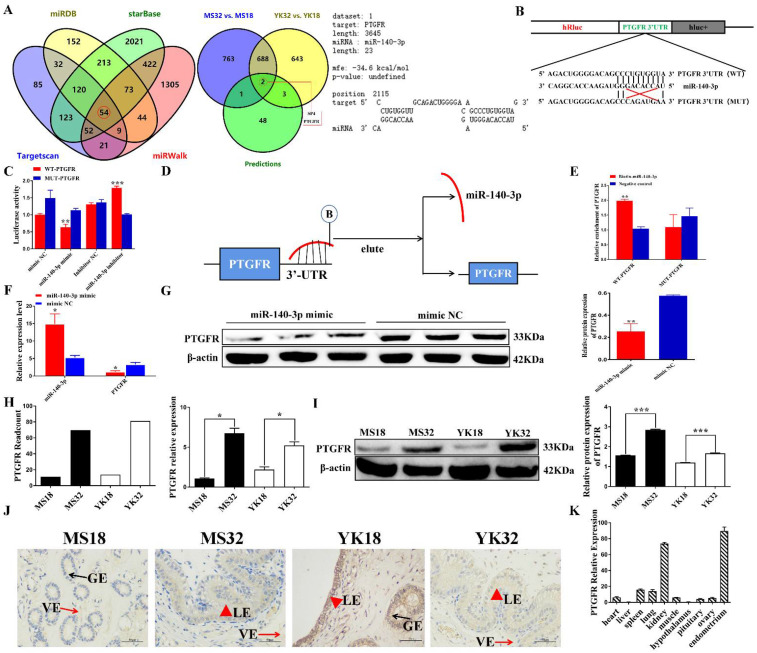
PTGFR acts as the functional target gene of miR-140-3p in EECs. (**A**) Venn plot showed the potential mRNAs of miR-140-3p according to multiple bioinformatics tools and RNA-seq data. (**B**) PTGFR protein functioned as the downstream target of miR-140-3p with complementary binding sites at the 3′ UTR. (**C**) Luciferase reporter assay validated the relative fluorescence intensity after the PTGFR 3′ UTR wild-type or mutant was co-transfected with miR-140-3p mimic or mimic NC and miR-140-3p inhibitor or inhibitor NC. (**D**) Schematic diagram displaying the pull-down of PTGFR caused by a biotin-labeled miR-140-3p probe. (**E**) RNA pulldown assay was performed with biotin-miR-140-3p, control or WT-PTGFR, or MUT-PTGFR using EECs extracts. The levels of miR-140-3p in immunoprecipitates were detected by qRT-PCR. (**F**) Overexpression of miR-140-3p down-regulated the PTGFR mRNA level in EECs. (**G**) Western blot showed the effect of overexpression of miR-140-3p on PTGFR protein expression. (**H**) The PTGFR mRNA levels in Yorkshire and Meishan endometrium at 18 and 32 days of pregnancy. (**I**) The protein levels of PTGFR in Yorkshire and Meishan endometrium at 18 and 32 days of pregnancy were detected by Western blot. (**J**) Immunohistochemical staining for PTGFR protein in endometrial tissue of Yorkshire and Meishan at 18 and 32 days of pregnancy. LE, luminal epithelium; GE, glandular epithelium; VE, vascular epithelium. (**K**) Detection of PTGFR expression level in 32-day pregnancy tissues Yorkshire pig by qRT-PCR. Error bars represent mean (n = 3) ± SD Student’s *t*-test; * *p* < 0.05, ** *p* < 0.01, *** *p* < 0.001.

Four prediction tools, starBase, miRWalk, miRDB, and TargetScan were used to predict the target genes for miR-140-3p and 54 target genes were filtered (Figure 5A; left panel) (Supplementary Text S3). Then, the target genes of miR-140-3p were intersected with genes differentially expressed in Meishan and Yorkshire pigs at different pregnancy stages, and Venn diagrams showed the intersected genes (Supplementary Text S4). Two intersected candidate target genes were screened (Figure 5A; middle panel). Notably, the target site of miR-140-3p predicted by RNAhybrid was found in the 3′UTR of porcine PTGFR (Figure 5A; right panel). 

To determine whether miR-140-3p directly targets porcine PTGFR through the predicted binding sites, the PTGFR 3′ UTR that contains the miR-140-3p targeted sites was cloned and inserted downstream of the luciferase gene in the psiCHECK-2 reporter plasmid. A mutant plasmid was constructed by inserting the mutant PTGFR 3′ UTR with the mutant binding site of miR-140-3p (Figure 5B). The wild-type (WT-PTGFR) or mutant plasmids (MUT-PTGFR) were co-transfected with the miR-140-3p mimic or mimic NC into PK15 cells. Luciferase activity was significantly lower in the miR-140-3p mimic group than the mimic NC group (*p* < 0.05), and this reduced activity was rescued in the mutant groups (Figure 5C). Moreover, an RNA pull-down assay with the biotin-labeled miR-140-3p probe was applied (Figure 5D). The results of qRT-PCR showed that the biotin-labeled miR-140-3p probe captured more WT-PTGFR than the negative control probe did. The biotin-labeled miR-140-3p probe cannot be enriched by MUT-PTGFR (Figure 5E). In addition, PTGFR mRNA and protein levels were significantly decreased after EECs were effectively transfected with miR-140-3p mimic (Figure 5F,G), whereas the miR-140-3p downregulation group showed opposite results (Supplementary Figure S3A,B). These data indicate that miR-140-3p can regulate PTGFR. 

PTGFR was a differentially expressed gene in comparing YK32 vs YK18 and MS32 vs MS18. The expression level of PTGFR was verified by qRT-PCR and Western blot (Figure 5H,I). The results revealed that PTGFR had a higher expression level in 32–day pregnant endometrium tissues. Immunohistochemical results showed that PTGFR protein was localized in the endometrial epithelium, including luminal and glandular epithelium (Figure 5J). Moreover, the expression levels of PTGFR in different tissues were also analyzed by qRT-PCR, and it is highest in the endometrium (Figure 5K). It can be concluded that PTGFR acts as the functional target of miR-140-3p in EECs.

### 3.6. MiR-140-3p Suppresses EECs Proliferation, Cycle Transition and Promotes Apoptosis by Targeting PTGFR 

**Figure 6:**
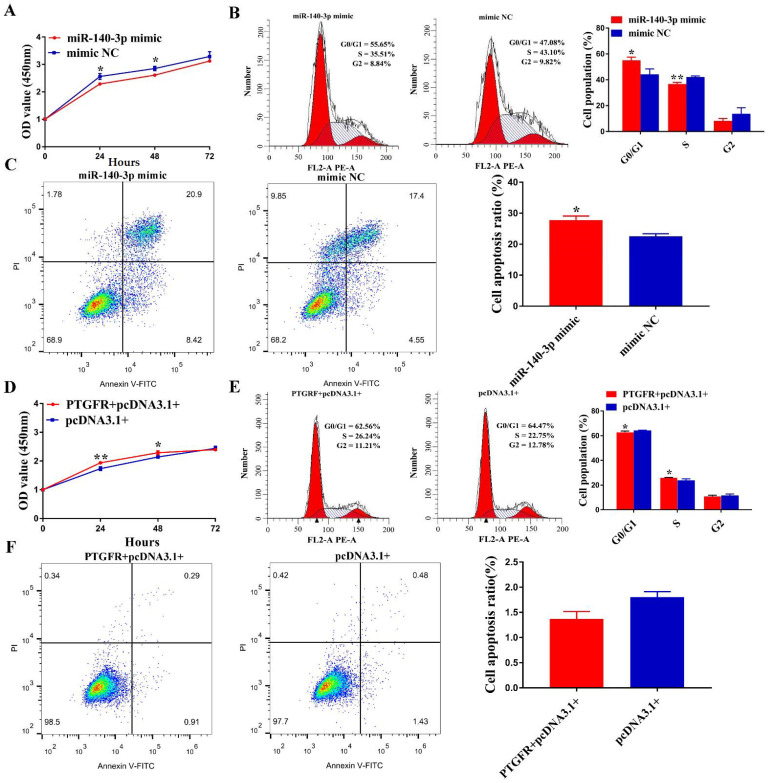
MiR-140-3p suppresses EECs proliferation, and cell cycle and induces apoptosis targeting PTGFR. (**A**) The proliferative ability of EECs transfected with miR-140-3p-mimic or mimic-NC was demonstrated by CCK-8. (**B**) Cell cycle progression was assessed by FACS assays in EECs transfected with miR-140-3p mimic or mimic NC. (**C**) The apoptosis rate of EECs after miR-140-3p mimic transfection was measured by FACS. (**D**) CCK-8 demonstrated the ability of EECs to proliferate with PTGFR or pcDNA3.1+ overexpression vectors. (**E**) The cell cycle PTGFR + pcDNA3.1 + transfected EECs was estimated by flow cytometry. (**F**) The apoptosis rate of EECs transfected with PTGFR and FACS determined pcDNA3.1 + overexpression vectors. Data are presented as mean ± SD of 3 subjects. * *p* < 0.05, ** *p* < 0.01.

Since few studies have investigated the role of miR-140-3p in EECs, we began to clarify the biological function of miR-140-3p in EECs. CCK-8 assay showed that miR-140-3p mimic suppressed the proliferative ability of EECs compared with mimic NC transfection (Figure 6A). In contrast, the proliferation of EECs was significantly enhanced when miR-140-3p was inhibited (Supplementary Figure S4A). In addition, FACS analysis results showed that miR-140-3p mimic led to more cells arrested in G0/G1 phase and less in S and G2/M phase (Figure 6B), whereas EECs were transfected with miR-140-3p inhibitor exerted the opposite role (Supplementary Figure S4B). Besides, the apoptosis rate of EECs transfected with miR-140-3p mimic or inhibitors was assessed by FACS assays. The results showed that the overexpression of miR-140-3p significantly increased EECs apoptosis (Figure 6C), while the miR-140-3p inhibitor showed the opposite effects (Supplementary Figure S4C). These experiments suggest that miR-140-3p suppresses EECs proliferation, and cycle transition and promotes apoptosis.

To further confirm the effect of PTGFR on the phenotype of EECs, PTGFR overexpression vectors and interfering fragments were transfected into EECs, respectively. CCK-8 assays demonstrated that the upregulation of PTGFR markedly enhanced the proliferation viability (Figure 6D), whereas si-PTGFR exerted the opposite function (Supplementary Figure S4D). An accelerated cell cycle can cause increased proliferation. Therefore, we measured the effect of PTGFR on cell cycle progression. After the effective overexpression of PTGFR, fewer EECs were arrested in the G0/G1 phase and accelerated into the S phase (Figure 6E). In contrast, EECs transfected with si-PTGFR showed the opposite result (Supplementary Figure S4E). PTGFR was hypothesized to regulate apoptosis of EECs because PTGFR silencing suppressed the survivability and proliferation of EECs. In contrast with the control groups, EECs that received PTGFR overexpression vector transfection had a significantly lower apoptosis ratio compared to the control group (Figure 6F), while the PTGFR silencing increased the ratio of apoptotic cells (Supplementary Figure S4F). These results demonstrated that miR-140-3p inhibits EECs proliferation, and cell cycle and induces apoptosis via binding to PTGFR.

### 3.7. Circ0001470 Promotes EECs Proliferation and Suppresses EECs Apoptosis through circ0001470/miR-140-3p/PTGFR Axis

**Figure 7:**
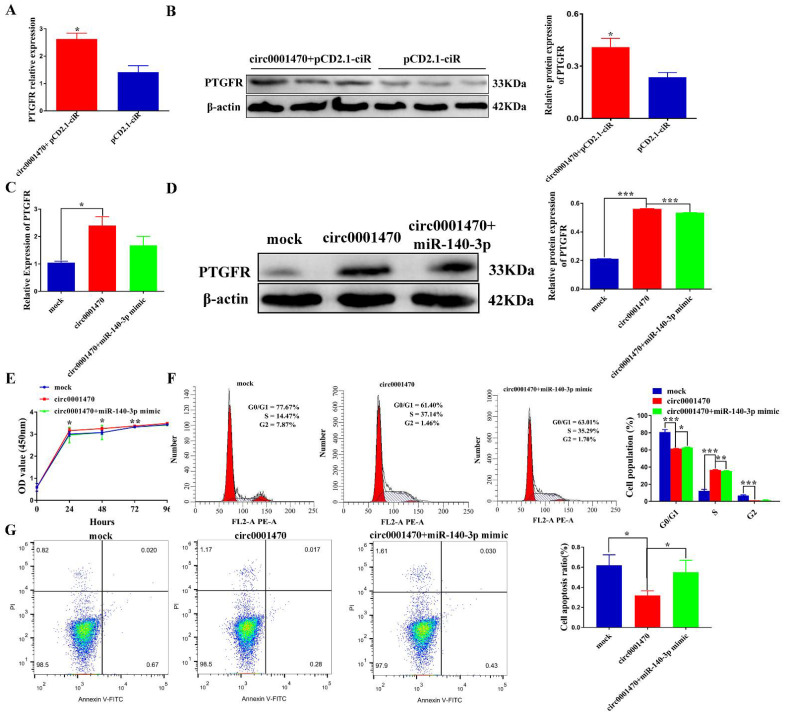
Circ0001470 functioned as a molecular sponge of miR-140-3p in EECs. (**A**) PTGFR mRNA levels in EECs overexpressed by circ0001470 were detected by qRT-PCR. (**B**) Western blot assays of the protein levels of PTGFR in the EECs with overexpression of circ0001470. (**C**) EECs were transfected with miR-140-3p mimic or circ0001470 overexpression vector, and qRT-PCR detected the relative expression levels of PTGFR. (**D**) Western blot detected the protein expression levels of PTGFR. (**E**) The cell viability was determined after transfection of indicated vectors, miR-140-3p mimic by CCK8 assay. (**F**) Cell cycle distribution of circ0001470 and/or miR-140-3p mimic transfected EECs was determined by flow cytometry. (**G**) The EECs apoptosis was determined by flow cytometry after being transfected with circ0001470 and/or miR-140-3p mimic. Data are presented as the mean ± SD for three subjects. * *p* < 0.05, ** *p* < 0.01, *** *p* < 0.001.

To further explore whether circ0001470 exerts its function by regulating the miR-140-3p/PTGFR pathway, several rescue experiments were carried out by co-transfection of miR-140-3p mimic or miR-140-3p inhibitor, and circ0001470 vector or si-circ0001470. Firstly, we transfected the overexpressed circ0001470 plasmid into EECs and found that it significantly increased the expression level of PTGFR using qRT-PCR and Western blot (Figure 7A,B). In addition, the expression level of PTGFR was significantly down-regulated after transfection of the circ0001470 interference fragment (Supplementary Figure S5A,B). Therefore, we can assume that the increases and decreases in PTGFR are induced by circ0001470 overexpression and knockdown, respectively. Secondly, to examine whether circ0001470 exerts its promoting function through miR-140-3p, the overexpression circ0001470 plasmid and miR-140-3p mimic were co-transfected into EECs. The results showed that the overexpression of circ0001470 significantly increased the expression of PTGFR according to qRT-PCR and Western blot. In contrast, the overexpression of miR-140-3p partially blocked the expression level of PTGFR (Figure 7C,D). In contrast, the inhibited effect of knockdown circ0001470 on PTGFR was significantly attenuated by miR-140-3p (Supplementary Figure S5C,D). Thus, these data suggest that circ0001470 influenced the PTGFR expression level of EECs by regulating the miR-140-3p level.

Rescue experiments were performed to understand whether circ0001470 affects the proliferation of EECs via miR-140-3p. The CCK-8 assays demonstrated that miR-140-3p mimic restrained the viability and proliferation of circ0001470-overexpressing EECs (Figure 7E). In contrast, miR-140-3p inhibitor restored viability and proliferation of EECs disrupted by circ0001470 (Supplementary Figure S5E). Moreover, flow cytometry analysis indicated that the overexpression of circ0001470 increased the ratio of cells in the S phase. However, this effect was partially reversed with the transfection of miR-140-3p mimic (Figure 7F). In contrast, the miR-140-3p knockdown could significantly restore the suppressive effects of circ0001470 downregulation on the EECs cycle (Supplementary Figure S5F). Moreover, flow cytometry analysis indicated that circ0001470 overexpression or miR-140-3p knockdown dramatically reduced apoptosis of EECs. Furthermore, miR-140-3p mimic triggered apoptosis was partially attenuated after co-transfection of circ0001470 overexpression vector (Figure 7G), whereas co-transfection of si-circ0001470 with miR-140-3p inhibitor, miR-140-3p inhibitor rescued si-circ0001470 from triggering apoptosis in EECs (Supplementary Figure S5G). These data hint that circ0001470 may act as a sponge for miR-140-3p, thus promoting EECs proliferation progression.

### 3.8. CircGRN Promotes Embryo Development In Vivo

**Figure 8:**
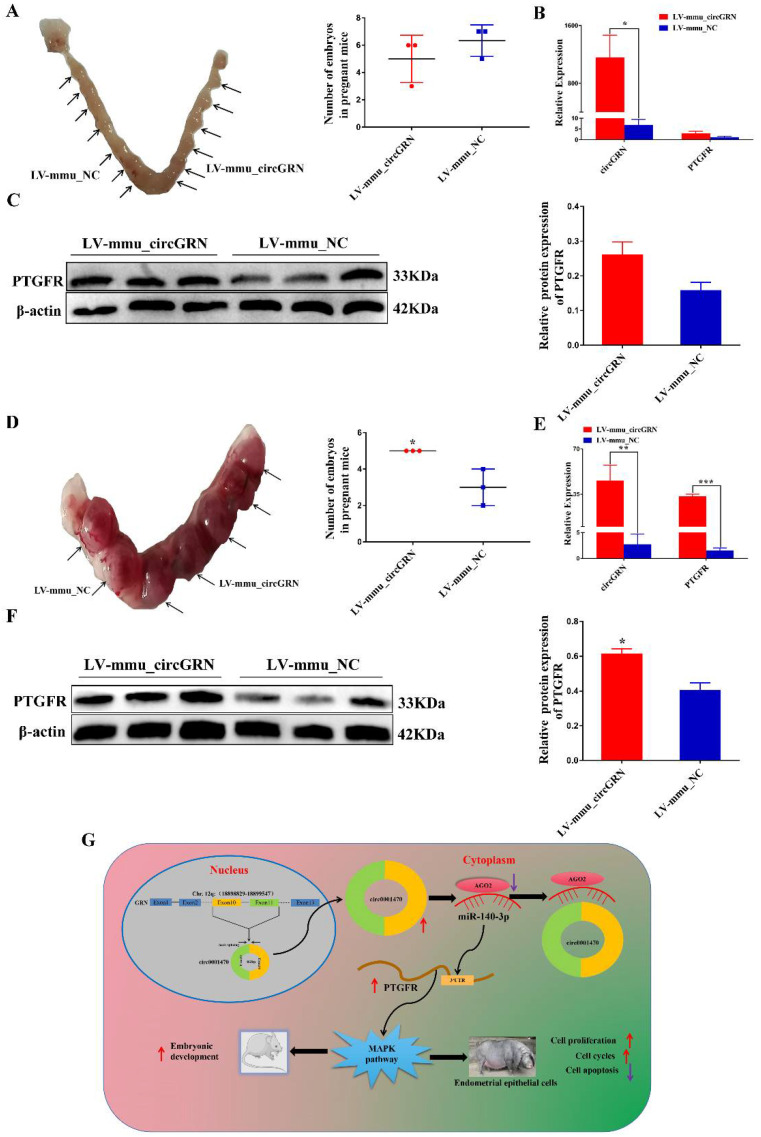
Analysis of mmu-circGRN function in mice during implantation and embryo development. (**A**) Comparison of the number of implantation sites between LV-mmu_NC injected uterine horn (**left**) and LV-mmu_circGRN injected uterine horn (**right**) on day five of pregnancy (n = 3). (**B**) The relative expression of circGRN and PTGFR in the pregnant mice embryos at the implantation stage (n = 3). (**C**) Relative expression levels of PTGFR proteins in the pregnant mice embryos at the implantation stage (n = 3). (**D**) Comparison of the number of embryonic development sites between LV-mmu_NC injected uterine horn (**left**) and LV-mmu_circGRN injected uterine horn (**right**) on day 10 of pregnancy (n = 3). (**E**) The relative expression levels of circGRN and PTGFR in the developing endometrium of pregnant mice (n = 3). (**F**) The relative expression levels of PTGFR proteins in the developing endometrium of pregnant mice (n = 3). (**G**) The hypothetical regulation model for circ0001470/miR-140-3p/PTGFR. Data are presented as the mean ± SD for three subjects. * *p* < 0.05, ** *p* < 0.01, *** *p* < 0.001.

To identify whether GRN produces a similar circRNA that also regulates embryo implantation and development in mice, we injected LV-mmu_circGRN and LV-mmu_NC plasmids into the right and left uterine horn of the mice respectively on day 0.5 of pregnancy, which has been known as the initiation of the pregnancy. qRT–PCR analysis identified that the level of LV-mmu_circGRN was dramatically increased in embryonic implantation stages. Interestingly, the results demonstrated that LV-mmu_circGRN had no significant effect on embryo numbers on day five of gestation compared with the LV-NC group (Figure 8A). Furthermore, the mRNA and protein expression levels of the target gene PTGFR did not achieve significant expression levels in vivo (Figure 8B,C). This might account for the different regulation patterns of circGRN during embryo implantation in mice and pigs. In vitro assays demonstrated that circ0001470 is associated with embryonic development. Therefore, we collected endometrial tissues at 10 days of pregnancy and counted the number of embryos. The results showed that the number of embryos in the LV-mmu_circGRN group was significantly higher than in the control group (Figure 8D). qRT–PCR analysis showed the level of LV-mmu_circGRN was dramatically increased during embryonic developmental stages. Furthermore, the mRNA and protein levels of PTGFR were significantly increased with the increased expression of circGRN (Figure 8E,F). These data suggest that mmu_circGRN may have an integral role in the regulation of embryonic development. Taken together, our study revealed that a novel circRNA named circ0001470 regulates porcine and murine embryo development progression through sponging miR-140-3p to regulate downstream PTGFR expression and active MAPK pathway (Figure 8G).

## 4. Discussion

In pigs, successful embryonic implantation and development require precise regulation between EECs and embryos [26], involving numerous molecular mediators [27]. Notably, recent studies showed that some lncRNAs could function as regulators in endometrium cells and be helpful for the endometrium-embryo crosstalk [28]. However, the functions of circRNA on EECs and on embryo development in pigs remain largely unknown. In this study, based on the high-throughput RNA-sequencing data, putative ceRNA networks were constructed by integrating circRNAs, miRNAs, and mRNAs in the endometrium of pigs. In spite of the limited endometrium samples, the major conclusion, the regulation of circ0001470 on embryo development, is unlikely to be significantly confounded [23,29]. To further apply circ0001470 to molecular genetic breeding, it is necessary to explore the effect of circ0001470 on the embryo development process.

During embryo implantation, the endometrium undergoes morphological and physiological changes, including cell proliferation and cell apoptosis [30,31]. The progression of the cell cycle commonly affects cell proliferation, and the breakdown of the cell cycle is regarded as a prevalent factor behind the repression of cell proliferation [32]. Several recent studies have reported that circRNAs regulate cell proliferation and apoptosis via binding to miRNAs in various cells. For instance, circLMO7 increased the ratio of bovine myoblasts cells in the S-phase of the cell cycle and protected them from apoptosis by binding to miR-378a-3p [33]. Additionally, circBTBD7 activates the p38 MAPK signaling pathway by regulating the miR-24-3p/MAPK7 axis, promoting proliferation and inhibiting apoptosis in immature porcine sertoli cells [34]. In this study, circ0001470 facilitated EECs proliferation and cell cycle, and the knockdown of circ0001470 induced EECs apoptosis. This suggests that circ0001470 might be involved in the regulation of embryo implantation by improving endometrial receptivity. The embryo number of day 32 pregnancy is regarded as one of the indexes of litter size in pigs. Circ0001470 is highly expressed in Meishan pigs during this period, which are known for high fecundity. Based on this, we speculate that there might be a positive correlation between circ0001470 and litter size in pregnant sows. Thus, we may identify litter size traits in early gestation based on the expression level of circ0001470, which can provide new ideas for the genetic improvement of pigs. In terms of embryo development, our study confirmed that the gain of function of LV-mmu_circGRN in mice promotes the initial development and survival of the embryo.

Previous studies suggested that quantitative highly expressed miRNAs were found in the endometrium of Meishan and Yorkshire pigs in early pregnancy [35,36]. MiR-140-3p involves various physiological processes by binding to circRNAs. For example, miR-140-3p is adsorbed by circRNA_000203, leading to enhanced Gata4 levels and thus exacerbating cardiac hypertrophy [37]. MiR-140-3p also suppressed breast cancer cell proliferation, migration, invasion, and glycolysis partially through the circ_0008039-miR-140-3p-SKA2 regulatory network [38]. In this study, miR-140-3p significantly increased during embryo implantation compared with the period of embryonic development, which was contrary to circ0001470. In addition, the effects of knockdown circ0001470 were suppressed by the miR-140-3p inhibitor in EECs. Combining dual luciferase and RNA pull-down assays, we confirmed that circ0001470 acted as a molecular sponge of miR-140-3p.

PTGFR can also regulate cell proliferation, apoptosis, and cell cycle in EECs. For instance, PTGFR signaling promotes the proliferation of EECs in cattle through the cell cycle [39]. PTGFR activation fosters the expression of PTGS-2 and growth factors via the activation of the PKC signaling pathway in bovine EECs [40]. Thus, we speculate that PTGFR may have a similar function in pigs. With Meishan and Yorkshire pigs as materials, our study found that PTGFR enhanced EECs proliferation, cycle transition, and inhibited apoptosis. Circ0001470 overexpression increased the mRNA and protein levels of PTGFR, while miR-140-3p mimic decreased PTGFR mRNA and protein levels in EECs. In addition, during murine embryo development, mmu_circGRN upregulated PTGFR expression, probably because the sequence of circGRN in mice is conservative with that of the porcine genome, leading to circGRN exerting its function in mice through the regulation of the ceRNA mechanism. These results further confirmed that circ0001470 acted as a ceRNA of miR-140-3p, and validated the function of PTGFR in porcine EECs. 

## 5. Conclusions

In conclusion, in pigs, circ0001470 promotes proliferation, and cycle transition and inhibits apoptosis of EECs, via a naturally expressed circ0001470-miR-140-3p-PTGFR network. Being highly sequentially conservative with circ0001470, mmu-circGRN also regulates the expression of PTGFR through a similar regulation pattern, and thus accelerates the embryonic development process. This may provide new insight into resolving mammalian reproduction problems.

## Data Availability

The data used to support the findings of this study are available from the corresponding author upon request.

## Supplementary Materials

The following supporting information can be downloaded at: https://www.mdpi.com/article/10.3390/cells11111746/s1, Figure S1: Cluster analysis of the number of differential expression circRNAs during the different periods of Meishan and Yorkshire pigs; Figure S2: Interference with Circ0001470 inhibits proliferation, cycling and promotes apoptosis of EECs; Figure S3: Knockdown of miR-140-3p up-regulated the PTGFR mRNA and protein levels in EECs; Figure S4: MiR-140-3p suppresses EEC cell proliferation, cycles and induces apoptosis targeting PTGFR; Figure S5: Interference with circ0001470 suppresses EECs proliferation, cycling and induces apoptosis through circ0001470/miR-140-3p/PTGFR axis. Table S1: The qRT-PCR primers used in the present study; Table S2: Primers for plasmid construction in the present study; Table S3: The sequence of pig circ0001470; Table S4: Oligonucleotides and probes used in this study; Table S5: List of antibodies used in this study; Table S6: The sequence of mouse circGRN. Text S1: Differential expression of circRNAs in endometrial tissues of Meishan and Yorkshire pigs at different gestation periods; Text S2: Predictive analysis of circ0001470 target miRNAs using circMine with differentially expressed miRNA-seq data from two breeds at different gestation periods; Text S3: Prediction of target genes for miR-140-3p by starBase, miRWalk, miRDB and TargetScan databases; Text S4: Bioinformatics screening of miR-140-3p target genes by combining two breeds of differentially expressed genes in endometrial tissue at different gestation periods.
