# Supplementary material for: Circ0001470 Acts as a miR-140-3p Sponge to Facilitate the Progression of Embryonic Development through Regulating PTGFR Expression
Source: Cells. 2022 May 25;11(11):1746. doi: 10.3390/cells11111746 (PMC9179393; doi:10.3390/cells11111746)
Supplement: Supplementary file 1 [file cells-11-01746-s001.zip › cells-1721071-supplementary.pdf]

Circ0001470 Acts as a miR-140-3p Sponge to Facilitate the Progression of Embryonic Development through Regulating PTGFR Expression

Long Zhang [1,2,3†], Changfan Zhou[1,2,3†], Xiaoyu Jiang[1,2,3], Shuntao Huang[1,2,3], Yiheng Li[1], Tao Su[1,2,3], Guowei Wang[1,2,3], You Zhou[1], Min Liu[1,4,] and Dequan Xu [1,2,3,]*

1 Colleges of Animal Science & Technology, Huazhong Agricultural University, Wuhan 430070,China; zlhzau.hzau.edu.cn@webmail.hzau.edu.cn (L.Z.); zhouchangfan@webmail.hzau.edu.cn (C.Z.); jxy.hzau.edu.cn@webmail.hzau.edu.cn (X.J.); shuntaohuang@webmail.hzau.edu.cn (S.H.); liyiheng@webmail.hzau.edu.cn(Y.L.); sutao@webmail.hzau.edu.cn (T.S.); wgw@webmail.hzau.edu.cn (G.W.); zhouyou_18@webmail.hzau.edu.cn (Y.Z.); liumin23@mail.hzau.edu.cn (M.L.)

2 Key Laboratory of Agricultural Animal Genetics, Breeding and Reproduction of Ministry of Education, Huazhong Agricultural University, Wuhan 430070, China

3 Key Laboratory of Swine Genetics and Breeding of Ministry of Agriculture and Rural Affairs, Huazhong Agricultural University, Wuhan 430070, China

4 College of Veterinary Medicine, Huazhong Agricultural University, Wuhan 430070, China

* Correspondence: dequanxu@126.com (D.X.)

† These authors equally contributed to this work.

Supplement material

Figure S1: Cluster analysis of the number of differential expression circRNAs during the different periods of Meishan and Yorkshire pigs

Figure S2: Interference with Circ0001470 inhibits proliferation, cycling and promotes apoptosis of EECs in vitro

Figure S3: Knockdown of miR-140-3p up-regulated the PTGFR mRNA and protein levels in EECs

Figure S4: MiR-140-3p suppresses EEC cell proliferation, cycles and induces apoptosis in vitro targeting PTGFR

Figure S5: Interference with circ0001470 suppresses EECs proliferation, cycling and induces apoptosis through circ0001470/miR-140-3p/PTGFR axis

Table S1: The qRT-PCR primers used in the present study

Table S2: Primers for plasmid construction in the present study

Table S3: The sequence of pig circ0001470

Table S4: Oligonucleotides and probes used in this study

Table S5: List of antibodies used in this study

Table S6. The sequence of mouse circGRN

ID	YK1801_readcount	YK1802_readcount	YK3201_readcount	YK3202_readcount	MS1801_readcount	MS1802_readcount	MS3201_readcount	MS3202_readcount
ssc_circ_0010224	0	0	0	66.50704975	324.3402624	122.4027663	0	0
ssc_circ_0013806	0	0	0	266.028199	442.282176	91.80207473	0	0
ssc_circ_0004860	71.16931179	0	0	66.50704975	147.427392	336.6076073	0	0
ssc_circ_0008331	249.0925913	0	0	266.028199	412.7966976	183.6041495	0	0
ssc_circ_0003658	177.9232795	0	73.52760962	299.2817239	176.9128704	367.2082989	0	0
ssc_circ_0004463	177.9232795	0	196.0736256	66.50704975	206.3983488	367.2082989	0	0
ssc_circ_0005797	249.0925913	0	0	0	117.9419136	581.4131399	0	0
ssc_circ_0009618	71.16931179	0	0	0	471.7676544	306.0069158	0	0
ssc_circ_0005428	320.2619031	0	0	0	412.7966976	183.6041495	0	0
ssc_circ_0009633	391.4312149	0	0	0	265.3693056	397.8089905	0	0
ssc_circ_0004857	0	2911.038659	0	931.0986965	2093.468967	734.4165978	0	0
ssc_circ_0009612	0	232.8830927	0	199.5211492	442.282176	489.6110652	0	0
ssc_circ_0011392	0	116.4415463	0	232.7746741	353.8257408	275.4062242	0	0
ssc_circ_0008036	0	174.6623195	73.52760962	0	442.282176	244.8055326	0	0
ssc_circ_0013046	0	291.1038659	147.0552192	0	855.0788737	673.2152147	0	0
ssc_circ_0007005	0	349.324639	0	0	383.3112192	581.4131399	0	0
ssc_circ_0004612	0	174.6623195	0	0	265.3693056	336.6076073	0	0
ssc_circ_0012608	0	232.8830927	0	0	383.3112192	459.0103736	0	0
ssc_circ_0009879	0	174.6623195	0	0	648.6805248	244.8055326	0	0
ssc_circ_0009213	0	407.5454122	0	0	530.7386112	306.0069158	0	0
ssc_circ_0011647	0	407.5454122	0	0	471.7676544	214.204841	0	0
ssc_circ_0000016	249.0925913	174.6623195	0	0	176.9128704	91.80207473	148.0092752	0
ssc_circ_0008193	533.7698384	261.9934793	0	0	0	550.8124484	0	0
ssc_circ_0005230	498.1851825	640.4285049	0	332.5352487	560.2240896	520.2117568	0	0
ssc_circ_0008789	1067.539677	1106.19469	0	465.5493482	1120.448179	1652.437345	0	0
ssc_circ_0012915	106.7539677	58.22077317	0	0	235.8838272	795.617981	0	0
ssc_circ_0005434	213.5079354	465.7661854	0	0	1208.904614	214.204841	0	0
ssc_circ_0009363	106.7539677	145.5519329	0	0	530.7386112	244.8055326	0	0
ssc_circ_0010296	106.7539677	174.6623195	0	0	294.854784	214.204841	0	0
ssc_circ_0004877	498.1851825	320.2142524	0	0	973.0207873	612.0138315	0	0
ssc_circ_0013353	213.5079354	116.4415463	0	0	353.8257408	214.204841	0	0
ssc_circ_0013095	355.846559	291.1038659	0	0	353.8257408	459.0103736	0	0
ssc_circ_0013443	1885.986763	1717.512809	0	0	2860.091405	1683.038037	0	0
ssc_circ_0001742	142.3386236	291.1038659	0	0	147.427392	153.0034579	0	0
ssc_circ_0010233	320.2619031	145.5519329	0	0	117.9419136	122.4027663	0	0
ssc_circ_0013072	71.16931179	407.5454122	73.52760962	99.76057462	0	0	148.0092752	0
ssc_circ_0013442	1138.708989	1164.415463	0	931.0986965	0	0	641.3735261	0
ssc_circ_0005279	604.9391502	873.3115976	245.0920321	99.76057462	117.9419136	122.4027663	148.0092752	0
ssc_circ_0001041	1174.293645	349.324639	392.1472513	0	0	0	0	0
ssc_circ_0012642	1031.955021	785.9804378	514.6932673	0	0	0	0	0

ssc_circ_0008932	177.9232795	203.7727061	122.546016	0	0	0	0	0
ssc_circ_0010210	177.9232795	203.7727061	98.03681282	0	0	0	0	0
ssc_circ_0000011	213.5079354	261.9934793	220.5828289	166.2676244	0	0	0	315.8005544
ssc_circ_0004695	213.5079354	203.7727061	269.6012353	99.76057462	0	0	0	105.2668515
ssc_circ_0010274	462.6005267	785.9804378	98.03681282	166.2676244	0	0	0	105.2668515
ssc_circ_0010668	6262.899438	69399.16162	3553.834465	4256.451184	0	0	0	105.2668515
ssc_circ_0007779	2348.587289	2416.162087	1715.644224	3125.831338	471.7676544	367.2082989	937.3920766	701.7790098
ssc_circ_0010218	498.1851825	145.5519329	882.3313154	365.7887736	0	0	0	0
ssc_circ_0006369	71.16931179	116.4415463	343.1288449	99.76057462	0	0	0	0
ssc_circ_0006669	71.16931179	145.5519329	245.0920321	232.7746741	0	0	0	0
ssc_circ_0010482	177.9232795	174.6623195	416.6564545	299.2817239	0	0	0	0
ssc_circ_0000813	106.7539677	378.4350256	98.03681282	66.50704975	0	0	0	0
ssc_circ_0000373	106.7539677	174.6623195	73.52760962	365.7887736	0	0	0	0
ssc_circ_0011419	249.0925913	174.6623195	122.546016	99.76057462	0	0	0	0
ssc_circ_0012139	355.846559	145.5519329	122.546016	133.0140995	0	0	0	0
ssc_circ_0002493	1423.386236	1164.415463	637.2392834	2394.253791	0	0	0	0
ssc_circ_0009323	427.0158708	436.6557988	245.0920321	332.5352487	0	0	0	0
ssc_circ_0007837	213.5079354	174.6623195	171.5644224	232.7746741	0	0	0	0
ssc_circ_0013441	1281.047612	1222.636237	759.7852994	1363.39452	0	0	0	0
ssc_circ_0010095	427.0158708	553.0973451	245.0920321	532.056398	0	61.20138315	0	0
ssc_circ_0001130	925.2010533	873.3115976	0	931.0986965	0	0	0	0
ssc_circ_0005282	320.2619031	87.33115976	0	66.50704975	0	0	0	0
ssc_circ_0001555	782.8624297	0	1225.46016	1396.648045	0	0	0	0
ssc_circ_0012483	391.4312149	0	245.0920321	332.5352487	442.282176	0	0	0
ssc_circ_0000841	0	145.5519329	98.03681282	332.5352487	206.3983488	0	0	0
ssc_circ_0011418	0	203.7727061	220.5828289	432.2958234	117.9419136	0	0	0
ssc_circ_0008663	782.8624297	1164.415463	245.0920321	498.8028731	1533.244877	0	0	0
ssc_circ_0007187	569.3544943	494.876572	490.1840641	299.2817239	117.9419136	0	0	0
ssc_circ_0000916	498.1851825	174.6623195	147.0552192	299.2817239	147.427392	0	0	0
ssc_circ_0010481	462.6005267	203.7727061	343.1288449	365.7887736	147.427392	0	0	0
ssc_circ_0011602	2562.095225	1833.954355	735.2760962	0	58.9709568	1162.82628	0	0
ssc_circ_0004874	106.7539677	58.22077317	73.52760962	0	678.1660032	428.4096821	0	0
ssc_circ_0010413	391.4312149	407.5454122	294.1104385	0	530.7386112	428.4096821	0	0
ssc_circ_0005426	676.108462	611.3181183	171.5644224	0	1356.332006	856.8193641	0	0
ssc_circ_0006333	391.4312149	261.9934793	245.0920321	0	1031.991744	826.2186725	0	0
ssc_circ_0011064	177.9232795	494.876572	612.7300801	598.5634477	471.7676544	183.6041495	0	105.2668515
ssc_circ_0001355	391.4312149	320.2142524	196.0736256	232.7746741	117.9419136	61.20138315	0	0
ssc_circ_0011616	7650.701018	12721.23894	1617.607412	1695.929769	707.6514816	856.8193641	0	0
ssc_circ_0009614	747.2777738	611.3181183	196.0736256	232.7746741	707.6514816	397.8089905	0	0
ssc_circ_0012525	1316.632268	756.8700512	392.1472513	232.7746741	796.1079169	642.6145231	0	0
ssc_circ_0001050	284.6772472	349.324639	49.01840641	99.76057462	235.8838272	306.0069158	0	0

ssc_circ_0002193	427.0158708	873.3115976	171.5644224	432.2958234	884.5643521	765.0172894	0	0
ssc_circ_0007268	177.9232795	203.7727061	98.03681282	99.76057462	265.3693056	367.2082989	0	0
ssc_circ_0007402	213.5079354	203.7727061	98.03681282	232.7746741	88.45643521	459.0103736	0	0
ssc_circ_0012514	142.3386236	58.22077317	49.01840641	133.0140995	235.8838272	397.8089905	0	0
ssc_circ_0001221	320.2619031	116.4415463	269.6012353	299.2817239	206.3983488	336.6076073	0	0
ssc_circ_0012140	355.846559	116.4415463	171.5644224	232.7746741	176.9128704	336.6076073	0	0
ssc_circ_0000579	1565.724859	582.2077317	784.2945026	598.5634477	796.1079169	1224.027663	0	0
ssc_circ_0001978	533.7698384	145.5519329	294.1104385	232.7746741	353.8257408	336.6076073	0	0
ssc_circ_0006038	213.5079354	378.4350256	171.5644224	432.2958234	353.8257408	520.2117568	0	0
ssc_circ_0010135	142.3386236	145.5519329	171.5644224	166.2676244	206.3983488	367.2082989	0	0
ssc_circ_0000251	284.6772472	261.9934793	367.6380481	332.5352487	619.1950464	428.4096821	0	0
ssc_circ_0012026	177.9232795	145.5519329	147.0552192	266.028199	353.8257408	306.0069158	0	0
ssc_circ_0012641	142.3386236	203.7727061	0	0	0	0	0	70.17790098
ssc_circ_0000449	106.7539677	320.2142524	0	0	0	0	0	0
ssc_circ_0012179	106.7539677	320.2142524	0	0	0	0	0	0
ssc_circ_0009305	320.2619031	349.324639	0	0	0	0	0	0
ssc_circ_0011615	1316.632268	1542.850489	0	0	0	0	0	0
ssc_circ_0011624	213.5079354	145.5519329	0	0	0	0	0	0
ssc_circ_0002488	355.846559	320.2142524	0	0	0	0	0	0
ssc_circ_0006269	213.5079354	174.6623195	0	0	0	0	0	0
ssc_circ_0013101	320.2619031	145.5519329	0	0	471.7676544	275.4062242	0	105.2668515
ssc_circ_0008443	640.5238061	378.4350256	0	0	294.854784	459.0103736	0	175.4447524
ssc_circ_0013428	391.4312149	145.5519329	0	0	117.9419136	367.2082989	0	140.355802
ssc_circ_0005424	1352.216924	582.2077317	0	133.0140995	1415.302963	1040.423514	0	140.355802
ssc_circ_0013665	3771.973525	1106.19469	0	66.50704975	1474.27392	3151.871232	98.67285017	245.6226534
ssc_circ_0005061	213.5079354	174.6623195	0	0	353.8257408	0	0	210.5337029
ssc_circ_0013646	320.2619031	232.8830927	0	0	412.7966976	0	0	315.8005544
ssc_circ_0002811	284.6772472	145.5519329	0	0	176.9128704	0	0	0
ssc_circ_0004114	391.4312149	291.1038659	0	0	324.3402624	0	0	0
ssc_circ_0013269	106.7539677	174.6623195	0	0	383.3112192	0	345.3549756	105.2668515
ssc_circ_0013487	249.0925913	0	0	0	235.8838272	0	444.0278257	385.9784554
ssc_circ_0011606	0	0	0	0	589.709568	0	345.3549756	596.5121583
ssc_circ_0000463	0	0	0	0	501.2531328	0	641.3735261	210.5337029
ssc_circ_0008518	0	0	0	0	471.7676544	0	493.3642508	315.8005544
ssc_circ_0001890	249.0925913	174.6623195	0	0	0	61.20138315	98.67285017	70.17790098
ssc_circ_0006069	213.5079354	291.1038659	0	0	0	428.4096821	246.6821254	175.4447524
ssc_circ_0007750	355.846559	261.9934793	0	0	0	214.204841	592.037101	491.2453069
ssc_circ_0006892	0	320.2142524	0	0	0	275.4062242	690.7099512	421.0674059
ssc_circ_0011214	0	320.2142524	0	0	265.3693056	306.0069158	246.6821254	456.1563564
ssc_circ_0007097	0	0	0	0	0	0	3996.250432	1543.913822
ssc_circ_0010582	0	0	0	0	0	0	444.0278257	245.6226534

ssc_circ_0006835	0	0	0	0	0	0	493.3642508	421.0674059
ssc_circ_0011452	0	0	0	0	0	0	1134.737777	1614.091723
ssc_circ_0004967	0	0	0	0	0	0	246.6821254	245.6226534
ssc_circ_0013119	0	0	0	0	0	0	345.3549756	350.8895049
ssc_circ_0012589	0	0	0	0	0	0	444.0278257	526.3342573
ssc_circ_0010601	0	0	0	0	0	0	838.7192264	912.3127127
ssc_circ_0011453	0	0	0	0	0	0	14356.8997	17228.67469
ssc_circ_0011238	6796.669276	3988.122962	6396.902037	5420.324554	10408.37388	13433.7036	26049.63244	18983.12221
ssc_circ_0001651	0	0	0	0	0	122.4027663	148.0092752	280.7116039
ssc_circ_0011224	1209.8783	931.5323707	661.7484866	964.3522213	1061.477222	1621.836654	2713.50338	2350.959683
ssc_circ_0001222	0	0	0	266.028199	265.3693056	244.8055326	296.0185505	0
ssc_circ_0007081	0	0	98.03681282	332.5352487	442.282176	275.4062242	148.0092752	0
ssc_circ_0000395	71.16931179	0	147.0552192	232.7746741	383.3112192	489.6110652	148.0092752	210.5337029
ssc_circ_0012340	0	0	73.52760962	66.50704975	147.427392	275.4062242	148.0092752	175.4447524
ssc_circ_0006851	0	0	122.546016	133.0140995	147.427392	244.8055326	148.0092752	175.4447524
ssc_circ_0013700	0	0	98.03681282	199.5211492	206.3983488	183.6041495	197.3457003	140.355802
ssc_circ_0002271	0	0	0	66.50704975	324.3402624	153.0034579	0	140.355802
ssc_circ_0012430	0	0	0	665.0704975	825.5933953	734.4165978	0	491.2453069
ssc_circ_0011269	0	0	0	232.7746741	324.3402624	367.2082989	641.3735261	631.6011088
ssc_circ_0011607	0	0	0	565.3099229	14064.5732	17289.39074	6167.053135	6386.188989
ssc_circ_0009597	0	87.33115976	147.0552192	0	589.709568	489.6110652	0	140.355802
ssc_circ_0010141	106.7539677	232.8830927	539.2024705	232.7746741	1326.846528	1101.624897	345.3549756	771.9569108
ssc_circ_0001285	0	0	147.0552192	0	147.427392	244.8055326	0	105.2668515
ssc_circ_0012691	0	0	122.546016	0	353.8257408	214.204841	0	456.1563564
ssc_circ_0004578	0	0	98.03681282	0	265.3693056	673.2152147	0	0
ssc_circ_0008068	0	0	98.03681282	0	265.3693056	214.204841	0	0
ssc_circ_0009698	0	0	122.546016	0	147.427392	244.8055326	0	0
ssc_circ_0006485	0	0	0	0	117.9419136	275.4062242	0	0
ssc_circ_0008969	0	0	0	0	206.3983488	397.8089905	0	0
ssc_circ_0011610	0	0	0	0	619.1950464	1071.024205	0	0
ssc_circ_0001399	0	0	0	0	383.3112192	214.204841	0	0
ssc_circ_0005410	0	0	0	0	825.5933953	397.8089905	0	0
ssc_circ_0003629	0	0	0	0	560.2240896	795.617981	0	0
ssc_circ_0013162	0	0	0	0	206.3983488	244.8055326	0	0
ssc_circ_0007172	0	0	0	0	589.709568	489.6110652	0	0
ssc_circ_0010758	0	0	0	0	265.3693056	275.4062242	0	0
ssc_circ_0005417	0	0	0	0	324.3402624	336.6076073	0	0
ssc_circ_0009629	0	0	0	0	294.854784	306.0069158	0	0
ssc_circ_0005594	213.5079354	0	0	0	678.1660032	275.4062242	493.3642508	280.7116039
ssc_circ_0010746	462.6005267	0	0	0	1120.448179	703.8159062	246.6821254	175.4447524
ssc_circ_0007370	0	0	0	0	176.9128704	214.204841	0	105.2668515

ssc_circ_0008330	0	0	0	0	265.3693056	336.6076073	0	140.355802
ssc_circ_0010215	391.4312149	291.1038659	294.1104385	365.7887736	3154.946189	1774.840111	789.3828013	1087.757465
ssc_circ_0013844	142.3386236	87.33115976	147.0552192	133.0140995	884.5643521	612.0138315	345.3549756	456.1563564
ssc_circ_0004891	0	0	269.6012353	0	206.3983488	428.4096821	296.0185505	456.1563564
ssc_circ_0005437	106.7539677	232.8830927	269.6012353	199.5211492	1031.991744	1591.235962	740.0463762	842.1348117
ssc_circ_0005961	0	0	0	0	353.8257408	306.0069158	394.6914007	140.355802
ssc_circ_0001063	0	0	0	0	265.3693056	306.0069158	197.3457003	210.5337029
ssc_circ_0011237	0	0	0	0	353.8257408	856.8193641	444.0278257	947.4016632
ssc_circ_0000980	0	0	294.1104385	232.7746741	206.3983488	0	0	0
ssc_circ_0006249	0	0	122.546016	133.0140995	383.3112192	91.80207473	0	0
ssc_circ_0008407	0	0	563.7116737	532.056398	88.45643521	0	0	210.5337029
ssc_circ_0009676	0	0	220.5828289	199.5211492	147.427392	0	0	140.355802
ssc_circ_0011170	0	0	98.03681282	266.028199	58.9709568	183.6041495	0	70.17790098
ssc_circ_0008318	0	0	294.1104385	133.0140995	117.9419136	122.4027663	0	140.355802
ssc_circ_0003131	0	0	220.5828289	232.7746741	235.8838272	214.204841	0	315.8005544
ssc_circ_0008819	0	0	98.03681282	133.0140995	176.9128704	214.204841	0	105.2668515
ssc_circ_0005258	0	0	122.546016	232.7746741	0	0	0	0
ssc_circ_0008109	0	0	122.546016	232.7746741	0	0	0	0
ssc_circ_0001188	0	0	269.6012353	332.5352487	0	0	0	0
ssc_circ_0011751	0	0	196.0736256	299.2817239	0	0	0	0
ssc_circ_0000984	0	0	1102.914144	1695.929769	0	0	0	0
ssc_circ_0005595	0	0	465.6748609	698.3240223	0	0	0	0
ssc_circ_0007087	0	0	686.2576898	332.5352487	0	0	0	0
ssc_circ_0008117	0	0	661.7484866	299.2817239	0	0	0	0
ssc_circ_0000986	0	0	171.5644224	166.2676244	0	0	0	0
ssc_circ_0008127	0	0	588.2208769	432.2958234	0	0	0	0
ssc_circ_0013283	213.5079354	349.324639	759.7852994	764.8310721	176.9128704	214.204841	98.67285017	105.2668515
ssc_circ_0012645	0	87.33115976	171.5644224	598.5634477	0	0	0	0
ssc_circ_0012670	0	58.22077317	98.03681282	399.0422985	0	0	0	0
ssc_circ_0007539	213.5079354	0	294.1104385	199.5211492	0	244.8055326	0	0
ssc_circ_0011271	0	0	147.0552192	266.028199	0	183.6041495	0	0
ssc_circ_0008245	71.16931179	0	196.0736256	465.5493482	0	0	148.0092752	350.8895049
ssc_circ_0013584	71.16931179	0	73.52760962	166.2676244	0	0	296.0185505	70.17790098
ssc_circ_0010987	320.2619031	0	465.6748609	332.5352487	0	0	641.3735261	456.1563564
ssc_circ_0013024	391.4312149	0	686.2576898	1064.112796	0	0	3206.86763	1508.824871
ssc_circ_0006980	320.2619031	232.8830927	416.6564545	166.2676244	0	91.80207473	740.0463762	561.4232078
ssc_circ_0003841	355.846559	1047.973917	955.858925	1729.183293	117.9419136	91.80207473	888.0556515	631.6011088
ssc_circ_0003843	355.846559	1047.973917	955.858925	1729.183293	117.9419136	91.80207473	888.0556515	631.6011088
ssc_circ_0009480	249.0925913	145.5519329	49.01840641	66.50704975	0	0	148.0092752	70.17790098
ssc_circ_0006490	71.16931179	116.4415463	147.0552192	133.0140995	0	0	296.0185505	140.355802
ssc_circ_0000998	391.4312149	203.7727061	245.0920321	332.5352487	0	0	789.3828013	456.1563564

ssc_circ_0003964	142.3386236	174.6623195	147.0552192	133.0140995	0	0	444.0278257	456.1563564
ssc_circ_0003837	284.6772472	116.4415463	490.1840641	199.5211492	0	0	345.3549756	315.8005544
ssc_circ_0012451	142.3386236	58.22077317	220.5828289	232.7746741	0	0	493.3642508	175.4447524
ssc_circ_0005118	0	58.22077317	49.01840641	0	0	0	296.0185505	175.4447524
ssc_circ_0005990	0	0	294.1104385	0	0	0	394.6914007	175.4447524
ssc_circ_0005110	106.7539677	87.33115976	73.52760962	0	0	0	148.0092752	245.6226534
ssc_circ_0010988	213.5079354	0	147.0552192	0	0	0	246.6821254	210.5337029
ssc_circ_0005197	0	0	465.6748609	1695.929769	88.45643521	0	1282.747052	1649.180673
ssc_circ_0008710	0	0	906.8405186	498.8028731	147.427392	0	690.7099512	1263.202218
ssc_circ_0008250	0	0	0	199.5211492	0	0	296.0185505	210.5337029
ssc_circ_0010603	0	0	0	299.2817239	0	0	2516.157679	1929.892277
ssc_circ_0003642	0	0	0	66.50704975	0	0	296.0185505	105.2668515
ssc_circ_0008129	0	0	0	199.5211492	0	0	789.3828013	245.6226534
ssc_circ_0009023	320.2619031	844.201211	539.2024705	1197.126895	973.0207873	581.4131399	2072.129853	3087.827643
ssc_circ_0009024	1281.047612	1833.954355	3039.141198	4888.268156	3538.257408	2815.263625	8535.201539	10386.32934
ssc_circ_0011873	71.16931179	0	441.1656577	532.056398	0	91.80207473	444.0278257	701.7790098
ssc_circ_0007090	0	0	1372.51538	1596.169194	0	612.0138315	2812.17623	1087.757465
ssc_circ_0007091	0	0	1544.079802	1030.859271	0	367.2082989	2664.166954	877.2237622
ssc_circ_0008112	0	0	2083.282272	1995.211492	0	61.20138315	2664.166954	491.2453069
ssc_circ_0009802	0	0	1789.171834	1296.88747	0	91.80207473	2170.802704	807.0458613
ssc_circ_0008114	0	0	9436.043234	7914.33892	0	183.6041495	5969.707435	2315.870732
ssc_circ_0009803	0	0	2083.282272	1695.929769	0	122.4027663	1677.438453	771.9569108
ssc_circ_0009804	0	0	1642.116615	1529.662144	0	214.204841	2664.166954	1754.447524
ssc_circ_0012672	0	0	490.1840641	997.6057462	0	61.20138315	641.3735261	842.1348117
ssc_circ_0012146	0	0	563.7116737	465.5493482	0	428.4096821	0	1087.757465
ssc_circ_0006739	0	0	196.0736256	565.3099229	0	0	0	280.7116039
ssc_circ_0008243	0	0	269.6012353	365.7887736	0	0	0	350.8895049
ssc_circ_0012763	0	320.2142524	3995.000123	2693.535515	0	367.2082989	5081.651784	1684.269623
ssc_circ_0006828	0	291.1038659	2450.920321	1529.662144	412.7966976	795.617981	1924.120578	1087.757465
ssc_circ_0006831	0	291.1038659	2205.828289	1330.140995	206.3983488	397.8089905	3206.86763	1157.935366
ssc_circ_0000981	0	0	7842.945026	8512.902368	0	0	542.7006759	0
ssc_circ_0000985	0	0	5808.68116	7482.043097	0	0	740.0463762	0
ssc_circ_0013569	0	0	294.1104385	66.50704975	0	0	197.3457003	0
ssc_circ_0012762	0	0	735.2760962	432.2958234	0	0	740.0463762	0
ssc_circ_0004301	0	0	416.6564545	166.2676244	0	0	493.3642508	0
ssc_circ_0008116	0	0	539.2024705	299.2817239	0	0	740.0463762	0
ssc_circ_0006494	854.0317415	1309.967396	2475.429524	2094.972067	530.7386112	428.4096821	5081.651784	3719.428752
ssc_circ_0000764	0	87.33115976	73.52760962	232.7746741	0	0	394.6914007	105.2668515
ssc_circ_0002146	0	58.22077317	196.0736256	232.7746741	0	0	345.3549756	175.4447524
ssc_circ_0000982	0	87.33115976	9950.736502	11738.49428	0	0	394.6914007	210.5337029
ssc_circ_0000983	0	0	10122.30092	11272.94493	0	0	394.6914007	210.5337029

ssc_circ_0005368	960.7857092	640.4285049	39999.01963	19353.55148	589.709568	2601.058784	32068.6763	17790.0979
ssc_circ_0008120	0	0	3112.668807	831.3381218	0	0	1825.447728	350.8895049
ssc_circ_0013368	0	0	269.6012353	166.2676244	0	0	197.3457003	70.17790098
ssc_circ_0007088	0	0	882.3313154	798.084597	0	0	592.037101	280.7116039
ssc_circ_0007089	0	0	490.1840641	565.3099229	0	0	394.6914007	175.4447524
ssc_circ_0008110	0	0	735.2760962	831.3381218	0	0	1085.401352	315.8005544
ssc_circ_0007095	0	0	955.858925	598.5634477	0	0	838.7192264	350.8895049
ssc_circ_0008111	0	0	784.2945026	565.3099229	0	0	1036.064927	350.8895049
ssc_circ_0004187	0	0	73.52760962	99.76057462	0	0	394.6914007	70.17790098
ssc_circ_0007094	0	0	539.2024705	565.3099229	0	0	1480.092752	315.8005544
ssc_circ_0008125	0	0	661.7484866	565.3099229	0	0	1578.765603	491.2453069
ssc_circ_0007093	0	0	12475.18443	13866.71987	0	0	28565.79012	14842.62606
ssc_circ_0008104	0	0	759.7852994	565.3099229	0	0	592.037101	561.4232078
ssc_circ_0002309	0	0	1715.644224	1230.38042	0	0	2318.811979	1228.113267
ssc_circ_0001470	0	0	269.6012353	232.7746741	0	0	296.0185505	175.4447524
ssc_circ_0013446	0	0	8945.85917	7083.000798	0	0	11643.39632	4807.186217
ssc_circ_0008124	0	0	3602.852871	1463.155094	0	0	7400.463762	2631.671287
ssc_circ_0011715	0	0	147.0552192	66.50704975	0	0	296.0185505	175.4447524
ssc_circ_0005207	5586.790976	7510.479739	38528.46744	41699.92019	1651.186791	3641.482297	35472.88963	82248.49995
ssc_circ_0006415	0	0	147.0552192	432.2958234	0	0	394.6914007	596.5121583
ssc_circ_0008248	0	0	269.6012353	1030.859271	0	0	444.0278257	771.9569108
ssc_circ_0005193	0	0	171.5644224	1030.859271	0	0	296.0185505	350.8895049
ssc_circ_0005201	0	0	73.52760962	931.0986965	0	0	197.3457003	385.9784554

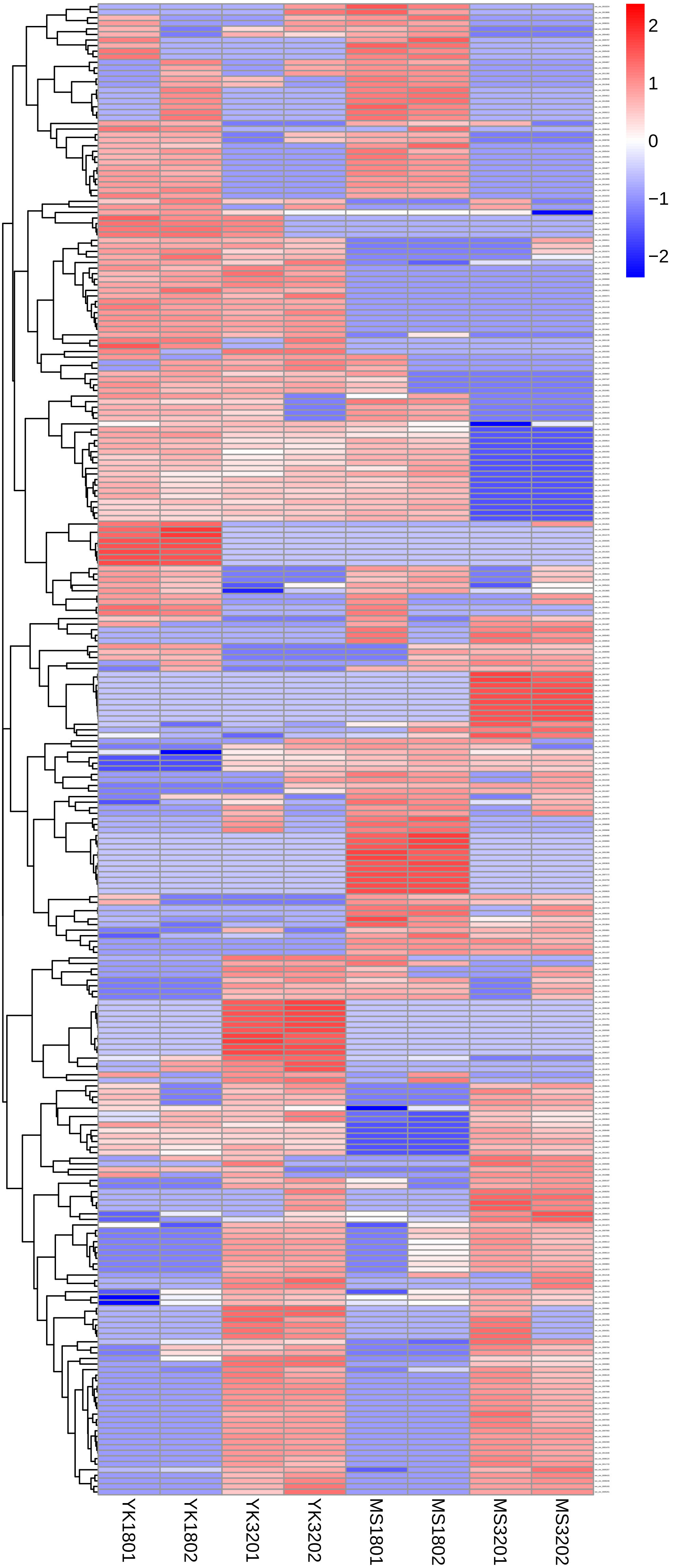

2
1
0
−1
−2
YK1801
YK1802
YK3201
YK3202
MS1801
MS1802
MS3201
MS3202

Figure S2: Interference with Circ0001470 inhibits proliferation, cycling and promotes apoptosis of EECs in vitro

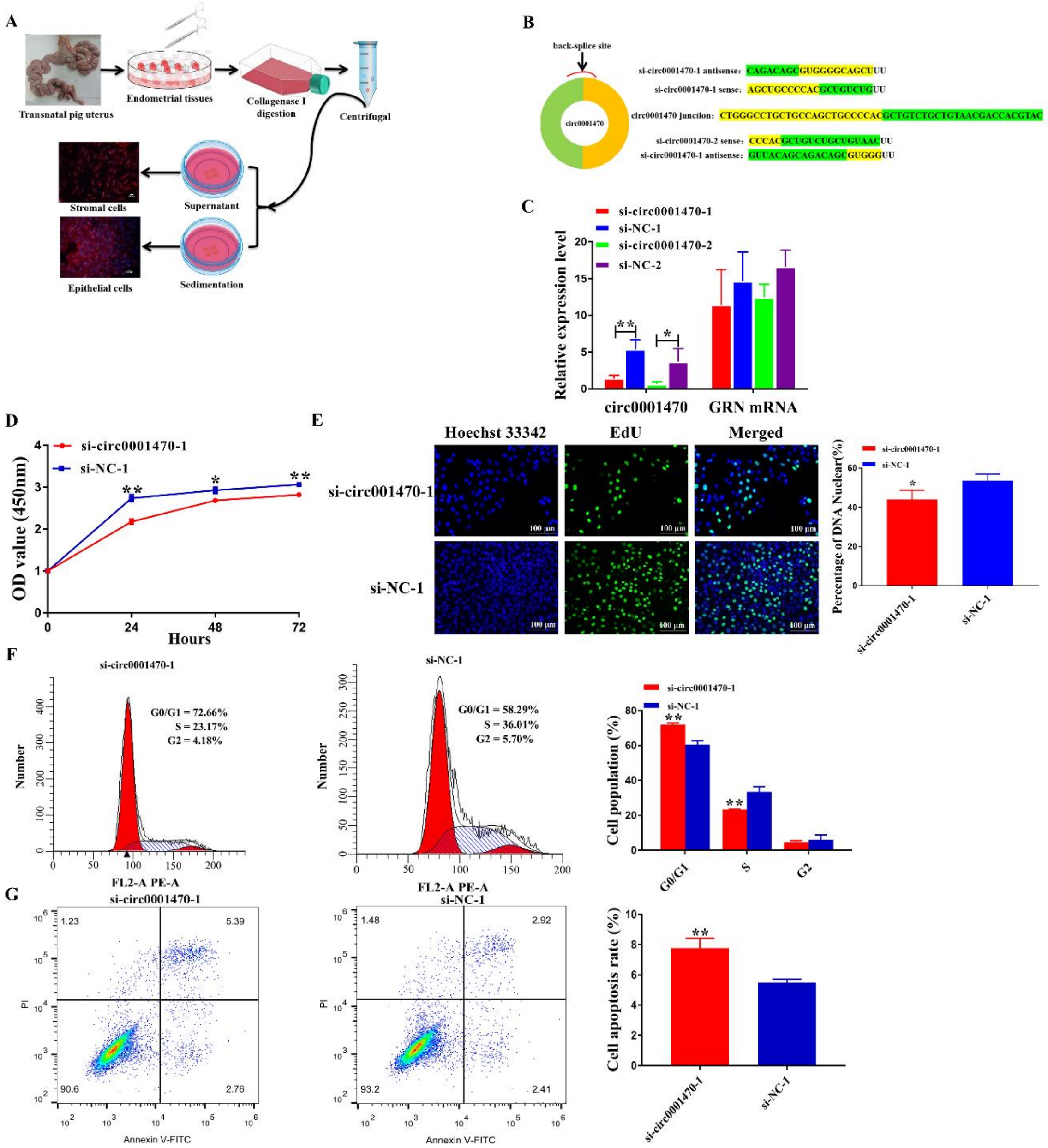

(A) The process of endometrial primary cell separation. **(B)** The schematic illustration showed circ0001470 specifically targeted the backsplice junction sequences of siRNAs. **(C)** qRT-PCR analysis of circ0001470 and GRN mRNA in EECs treated with siRNAs. **(D)** The proliferative ability of EECs transfected with si-circ0001470-1 or si-NC-1 was measured by CCK-8. **(E)** After transfection of si-circ0001470-1, the EdU method was used to detect cell proliferation. EdU staining (green) for positive cells. Scale bars, 100 μm. **(F)** Cell cycle distribution was measured in EECs transfected with si-circ0001470-1

or si-NC-1. **(G)** FCM analysis showed the ratio of apoptotic EECs following the treatments of si-circ0001470-1 and si-NC-1. Data were shown as mean ± SD; All in vitro data are representatives of three independent experiments. *P < 0.05, **P < 0.01.

Figure S3: Knockdown of miR-140-3p up-regulated the PTGFR mRNA and protein levels in EECs

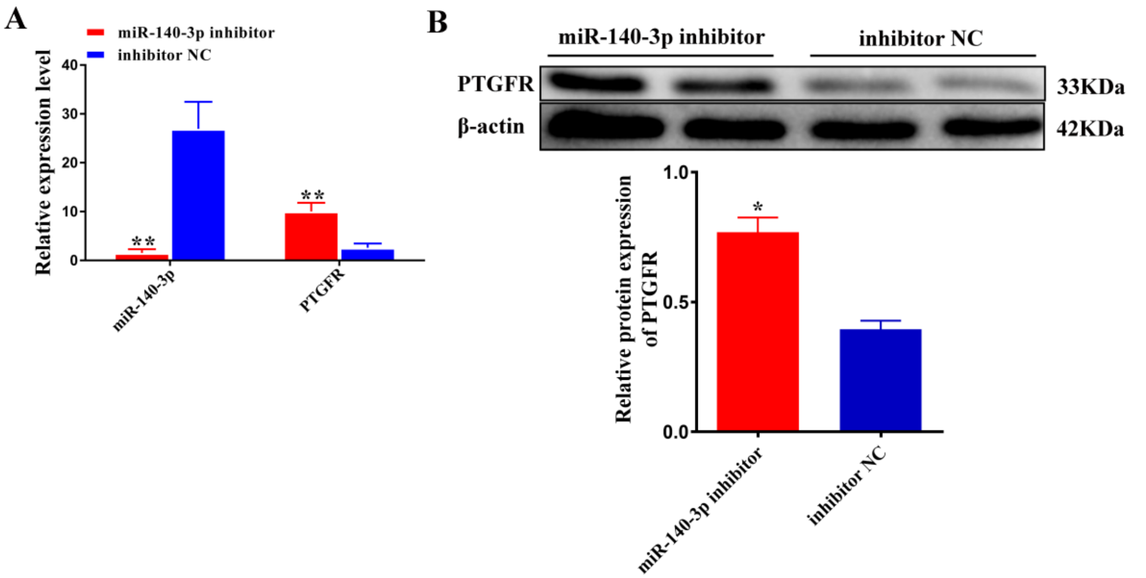

(A) qRT-PCR assays for miR-140-3p interference efficiency and effect on the expression level of the target gene PTGFR. **(B)** The effect of down-regulation of miR-140-3p on PTGFR protein expression levels as determined by western blot. Data are presented as mean ± standard deviation *P < 0.05, **P < 0.01.

Figure S4: MiR-140-3p suppresses EEC cell proliferation, cycles and induces apoptosis in vitro targeting PTGFR

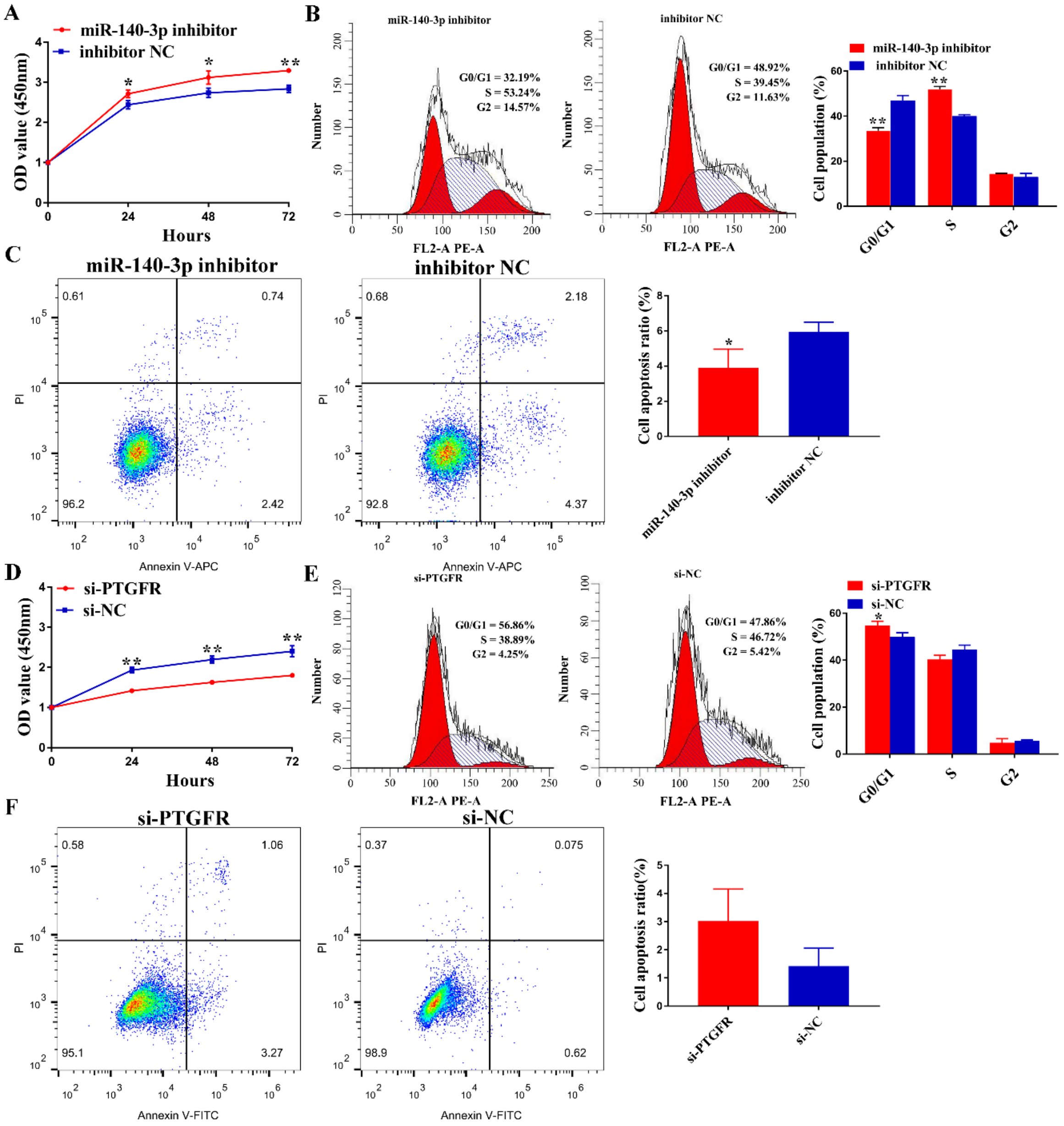

(A) The cell proliferation was detected by CCK-8 assay after transfection of miR-140-3p inhibitor. **(B)** EECs were transfected with miR-140-3p inhibitor, and the cell cycle was analyzed by flow cytometry. **(C)** The apoptosis rate was assessed by V-FITC/PI annexin staining and flow cytometry. **(D)** The cell viability was measured in EECs transfected with si-PTGFR or si-NC by CCK-8 assay. **(E)** The cell cycle progression was analyzed by flow cytometry after transfected with si-PTGFR or si-NC. **(F)** The apoptosis rate detected by flow cytometry after downregulation of PTGFR. Data are presented as mean ± standard deviation $*P < 0.05$, $**P < 0.01$.

Figure S5: Interference with circ0001470 suppresses EECs proliferation, cycling and induces apoptosis through circ0001470/miR-140-3p/PTGFR axis

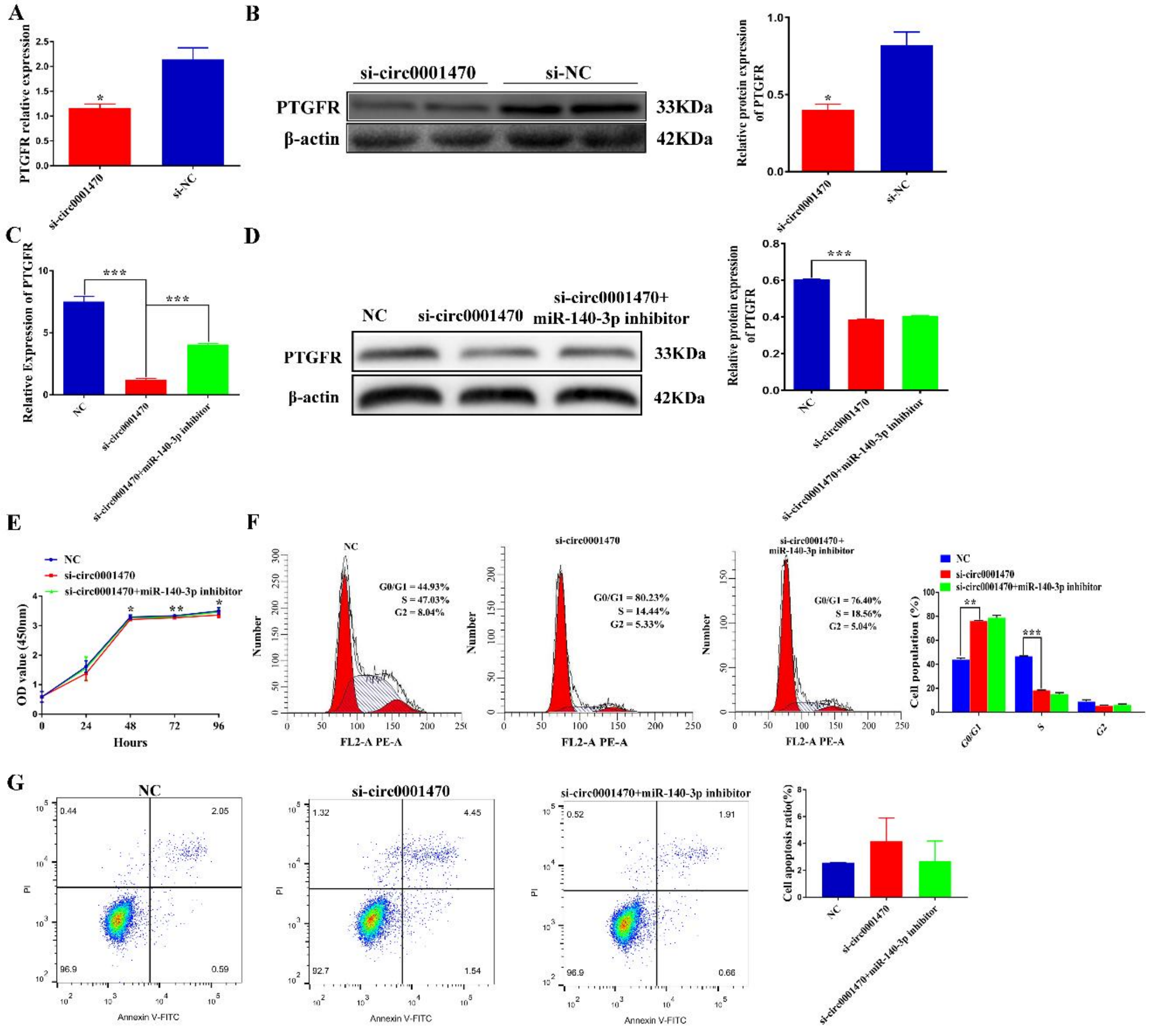

(A) qRT-PCR detected the mRNA levels of PTGFR in the EECs with the knockdown of circ0001470. **(B)** Western blot detected the protein levels of PTGFR in the EECs with the knockdown of circ0001470. **(C)** The relative mRNA levels of PTGFR in EECs transfected with miR-140-3p inhibitor, NC or si-circ0001470 were measured by qRT-PCR assay. **(D)** The relative protein levels of PTGFR in EECs transfected with indicated inhibitor, NC or si-circ0001470 were measured by western blot assay. **(E)** The proliferative ability of EECs transfected with NC or si-circ0001470 was assessed by CCK-8. **(F)** Flow cytometry assay demonstrated that the effects on EECs cycle caused by si-circ0001470 was partially rescued by miR-140-3p inhibitor. **(G)** Flow cytometry assay assessed cell apoptosis in EECs transfected with different constructs. Data are presented as mean ± standard deviation *P < 0.05, **P < 0.01, ***P < 0.001.

Table S1: The qRT-PCR primers used in the present study

Name	Primer	Sequence (5'-3')
pig circ0001470 (qPCR)	Forward primer	AAACACCACGGGCTGTGACCA
	Reverse primer	CTTCTCTATGTTACACTGATA
mouse circ0001470 (qPCR)	Forward primer	TGGAGATATCGGTTGTGACCA
	Reverse primer	ACCCCTGAGCCAGACATGTGA
circ0001470 (Junction sites)	Forward primer	CTGCCCCACGCTGTCTGCTGTA
	Reverse primer	ACGTTATCACAGGGGACATCTCT
linear GRN	Forward primer	CAGTGGGTGCCCTGGAAGGAGAA
	Reverse primer	ACGTGTGGCCCTTGGGGCAGCA
β-actin	Forward primer	CTCCATCATGAAGTGCGACGT
	Reverse primer	GTGATCTCCTTCTGCATCCTGTC
miR-140-3p	Stem-loop Primer	CTCAACTGGTGTCGTGGAGTC GGCAATTCAGTTGAGGTCCGTGG
	Forward primer	TCGGCAGGTACCACAGGGTAGAAC
	Reverse primer	CTCAACTGGTGTCGTGGAGT
U6	Forward primer	GCTTCGGCAGCACATATACT
	Reverse primer	TTCACGAATTTGCGTGTCAT
pig PTGFR	Forward primer	GATGATGTTGAGTGGCGCGT
	Reverse primer	CCCGTGATGGCATTGCAAAA
mouse PTGFR	Forward primer	AATGATCCTGAGTGGTGTGT
	Reverse primer	ACTCCCGTGACGGCATTGCAC

Table S2: Primers for plasmid construction in the present study

Name	Primer	Sequence (5'-3')
circ0001470(pCD2.1-ciR)	Forward primer	GGGGTACCCCGCTGTCTGCTGTAACGACCACG
	Reverse primer	CGGCTAGCCGGTGGGGCAGCTGGCAGCAGGCC
circ0001470(pCD2.1-ciR)-mut	Forward primer	GAGAGATGTCCCCTACTATAAACGTCTCCAGCTGTCCCTCCT
	Reverse primer	TTATAGTAGGGGACATCTCTCTCCATGGCTAGGAGGTC
circ0001470(psiCHECK-2-WT)	Forward primer	CCCTCGAGGGGCTGTCTGCTGTAACGACCACG
	Reverse primer	ATTTGCGGCCGCTTTAGTGGGGCAGCTGGCAGCAGGCC
circ0001470(psiCHECK-2-MUT)	Forward primer	GAGAGATGTCCCCTACTATAAACGTCTCCAGCTGTCCCTCCT
	Reverse primer	TTATAGTAGGGGACATCTCTCTCCATGGCTAGGAGGTC
PTGFR(pcDNA3.1+)	Forward primer	CGGAATTCCGGGCCAGCTCTTACTCCACAA
	Reverse primer	CCCTCGAGGGCCAGTTAGGCTTTTTGCATGTCT
PTGFR(psiCHECK-2-WT)	Forward primer	CCCTCGAGGGTGTTCTCATGTGTTTTAGGG
	Reverse primer	ATTTGCGGCCGCTTTATCCTGTACATGATCATGTCACA
PTGFR(psiCHECK-2-MUT)	Forward primer	TGAACATGCCTACTATTGCAGACTGGGGACAGCCCTG
	Reverse primer	ATAGTAGGCATGTTCACGCAGCATCCTGCCCCACATGGG

Table S3: The sequence of pig circ0001470

>circ0001470
GCTGTCTGCTGTAACGACCACGTACACTGCTGCCCGACCGGATATCAGTGTA
ACATAGAGAAGGGCACCTGTGACCTGGAGACCCAGTGGGTGCCCTGGAAGG
AGAAGATCCCGGCCAGCCTCAGCCGGCCGGACCTCCTAGCCATGGAGAGAG
ATGTCCCCTGTGGTAACGTCTCCAGCTGTCCCTCCTCCAGTACCTGCTGTCCA
GTCACGCCTGGAGAGTGGGGCTGCTGTCCTGCCCCAGAGGCTGTCTGCTGCT
CGGACCACCAGTACTGCTGCCCCAAGGGCCACACGTGTGTCGGCAAGGGACA
CTGTAAGAGGAAGAAGGACATGGTGACCGGACTGAACAAGATGCCCACCCG
CCGGGCTTCCGCGTCCCAGCCCGGAAACACCACGGGCTGTGACCAGCACACC
AGTTGCCCGGTGGGGCAGACCTGCTGCCCGAGCCTGAGCAAGGGCTGGGCCT
GCTGCCAGCTGCCCCAC

Table S4: Oligonucleotides and probes used in this study

Definition	Sequence (5'-3')
si-circ0001470-1	Sense: AGCUGCCCCACGCUGUCUGUU<br>Antisense: CAGACAGCGUGGGGCAGCUUU
si-circ0001470-2	Sense: CCCACGCUGUCUGCUGUAACUU<br>Antisense: GUUACAGCAGACAGCGUGGGUU
si-circ0001470 NC	Sense: UUCUCCGAACGUGUCACGUTT<br>Antisense: ACGUGACACGUUCGGAGAATT
miR-140-3p mimic	UACCACAGGGUAGAACCACGGAC
miR-140-3p mimic NC	UCACAACCUCCUAGAAAGAGUAGA
miR-140-3p inhibitor	GUCCGUGGUUCUACCCUGUGGUA
miR-140-3p inhibitor NC	UCUACUCUUUCUAGGAGGUUGUGA
si-PTGFR-1	Sense: AGAAAUCUGUGAUUACUAGGG<br>Antisense: CUAGUAAUCACAGAUUUCUUU
si-PTGFR-2	Sense: AUUCGAAGAGUAAAAAGGGUU<br>Antisense: CCCUUUUUACUCUUCGAAUGG
si-PTGFR NC	Sense: UUCUUCGAACGUGUCACGUTT<br>Antisense: ACGUGACACGUUCGGAGAATT
Cy5-labeled circ0001470 probe	aaaCGTTACAGCAGACAGCGTGGGGCAGCTGGCAG
Biotin miR-140-3p probe	aaaGTCCGTGGTTCTACCCTGTGGTA
FAM-labeled miR-140-3p probe	aaaGTCCGTGGTTCTACCCTGTGGTA

Table S5: List of antibodies used in this study

Name	Company	Catalog Number
Anti-PTGFR	Abcam	ab188935
Anti-AGO2	Cell Signaling Technology	#2897
Anti-β-actin	ABclonal	AC038
Goat Anti-Rabbit IgG H&L (HRP)	ABclonal	AS014

Table S6. The sequence of mouse circGRN

>circGRN
GCCGTGTGTTGTGAGGATCACATTCATTGCTGCCCGGCAGGGTTTCAGTGTCA
CACAGAGAAAGGAACCTGCGAAATGGGTATCCTCCAAGTACCCTGGATGAAG
AAGGTCATAGCCCCCCTCCGCCTGCCAGACCCACAGATCTTGAAGAGTGATA
CACCTTGTGATGACTTCACTAGGTGTCCTACAAACAATACCTGCTGCAAACTC
AATTCTGGGGACTGGGGCTGCTGTCCCATCCCAGAGGCTGTCTGCTGCTCAGA
CAACCAGCATTGCTGCCCTCAGGGCTTCACATGTCTGGCTCAGGGGTACTGTC
AGAAGGGAGACACAATGGTGGCTGGCCTGGAGAAGATACCTGCCCGCCAGA
CAACCCCGCTCCAAATTGGAGATATCGGTTGTGACCAGCATACCAGCTGCCC
AGTAGGGCAAACCTGCTGCCCAAGCCTCAAGGGAAGTTGGGCCTGCTGCCAG
CTGCCCCAT
